# Development of a nanoparticle-based immunotherapy targeting PD-L1 and PLK1 for lung cancer treatment

**DOI:** 10.1038/s41467-022-31926-9

**Published:** 2022-07-23

**Authors:** Moataz Reda, Worapol Ngamcherdtrakul, Molly A. Nelson, Natnaree Siriwon, Ruijie Wang, Husam Y. Zaidan, Daniel S. Bejan, Sherif Reda, Ngoc Ha Hoang, Noah A. Crumrine, Justin P. C. Rehwaldt, Akash Bindal, Gordon B. Mills, Joe W. Gray, Wassana Yantasee

**Affiliations:** 1 PDX Pharmaceuticals, Inc., Portland, OR USA; 2 Department of Biomedical Engineering, Oregon Health and Science University, Portland, OR USA; 3 Department of Cell, Developmental & Cancer Biology, Oregon Health and Science University, Portland, OR USA; 4 Knight Cancer Institute, Oregon Health and Science University, Portland, OR USA

**Keywords:** Nanoparticles, Cancer immunotherapy, Non-small-cell lung cancer

## Abstract

Immune checkpoint inhibitors (ICIs) targeting PD-L1 and PD-1 have improved survival in a subset of patients with advanced non-small cell lung cancer (NSCLC). However, only a minority of NSCLC patients respond to ICIs, highlighting the need for superior immunotherapy. Herein, we report on a nanoparticle-based immunotherapy termed ARAC (Antigen Release Agent and Checkpoint Inhibitor) designed to enhance the efficacy of PD-L1 inhibitor. ARAC is a nanoparticle co-delivering PLK1 inhibitor (volasertib) and PD-L1 antibody. PLK1 is a key mitotic kinase that is overexpressed in various cancers including NSCLC and drives cancer growth. Inhibition of PLK1 selectively kills cancer cells and upregulates PD-L1 expression in surviving cancer cells thereby providing opportunity for ARAC targeted delivery in a feedforward manner. ARAC reduces effective doses of volasertib and PD-L1 antibody by 5-fold in a metastatic lung tumor model (LLC-JSP) and the effect is mainly mediated by CD8+ T cells. ARAC also shows efficacy in another lung tumor model (KLN-205), which does not respond to CTLA-4 and PD-1 inhibitor combination. This study highlights a rational combination strategy to augment existing therapies by utilizing our nanoparticle platform that can load multiple cargo types at once.

## Abstract (web summary)

Only a minority of patients with non-small cell lung cancer (NSCLC) respond to immune checkpoint inhibitors. Here the authors design a nanosystem for the co-delivery of a PLK1 inhibitor and PD-L1 antibody, showing anti-tumor immune responses in preclinical lung cancer models.

## Introduction

The emergence of immune checkpoint inhibitors (ICIs), specifically antibodies targeting the PD-1/PD-L1 axis, has reshaped the treatment landscape for various cancers including non-small cell lung cancer (NSCLC). PD-L1 expression on tumor cells inhibits tumor-directed cytotoxic CD8+ T cell activity by binding to PD-1 receptors on T cells and suppressing their function^1–3^. Thus, PD-1/PD-L1 overexpression is a hallmark of the immunosuppressive environment displayed in several types of cancer. PD-1/PD-L1 blockade induces potent responses in patients; however, only 15-20% of patients respond, and many initial responders often relapse suggesting resistance mechanisms^4–6^. PD-L1 expression has emerged as a biomarker to predict response to ICIs as high levels of PD-L1 are often associated with better treatment outcomes^7^, while low levels of tumor infiltrating lymphocytes (TILs) are associated with lack of response or eventual resistance^7,8^. Thus, combinatorial strategies employing PD-L1/PD-1 inhibitors with other therapeutics that (1) upregulate PD-L1 expression and/or (2) increase the density of TILs have great potential to enhance the response rate of treatments, and identify more curative approaches. In addition to low clinical response rate, systemic distribution of ICIs can result in pathologic autoimmunity, leading to immune-related adverse events (irAEs) that damage normal tissues^9^. Therefore, strategies to localize treatment effects to the tumor and thus improve safety have important clinical relevance.

Polo-like kinase 1 (PLK1) is a critical mitotic kinase that is overexpressed in various cancers and provokes oncogenic properties^10^. Previous studies have illustrated the potential of PLK1 inhibition as a strong therapeutic strategy, and several PLK1 small molecule inhibitors have reached clinical trials^11^. However, clinical utility of PLK1 inhibitors has yet to be realized due to dose-limiting toxicities and poor efficacy as a monotherapy; thus, alternative therapeutic strategies are needed to elicit the full potential of PLK1 inhibition. In addition to its onco-proliferative functions, PLK1 also has roles in immune regulation in several types of cancer^12^. In NSCLC, PLK1 expression is negatively correlated with immune scores, major histocompatibility complex (MHC) class I activity, and expression of TILs^12^. PLK1 expression has also been shown to activate STAT3, which promotes an immunosuppressive tumor microenvironment (TME)^13^. Inhibition of PLK1 results in reduced phosphorylation of STAT3^14,15^, thereby dampening its activity in NSCLC cells. Furthermore, PLK1 inhibition has been shown to increase MHC class I expression in multiple cell lines^12^, suggesting a role in adaptive immunity. In short, PLK1 expression contributes to immunosuppressive TME, inhibiting immune cell infiltration and anti-tumor immunity. Thus, in addition to direct toxicity in cancer cells, inhibiting PLK1 may enhance anti-tumor immunity and synergize with immunotherapy.

In this work, we investigate the relationship of PLK1 inhibition with immune checkpoint blockade. We report that PLK1 inhibition upregulates PD-L1 expression in cancer cells, thereby diminishing cytotoxic T cell function. Accordingly, the combination of PD-L1 blockade with PLK1 inhibition significantly reduces tumor burden and prolongs survival of lung tumor-bearing mice. To facilitate clinical translation of this combination, we develop a nano-immunotherapy termed ARAC (Antigen Release Agent and Checkpoint inhibitor) for co-delivery of a PLK1 inhibitor (volasertib) and PD-L1 antibody. ARAC is built upon our polymer-coated mesoporous silica nanoparticle (NP) platform, which has shown substantial promise as a tumor-targeted delivery vehicle^14,16–18^. Indeed, we show that delivery of the therapeutic compounds (PLK1 inhibitor (volasertib) and PD-L1 antibody) with our NP platform can reduce the effective drug doses by 5-fold in an aggressive metastatic lung tumor model. This is significant as it alleviates toxicity concerns of each drug, while achieving the therapeutic synergy of the drug combination. PLK1 inhibition induces cancer cell death while also upregulating PD-L1 expression of surviving cancer cells, thereby providing the opportunity to target surviving cancer with PD-L1 antibody-conjugated nanoparticles (benefiting cancer otherwise having no established receptors for drug delivery). The upregulation of PD-L1 expression also renders cancer more responsive to PD-1/PD-L1 blockade with PD-L1 antibody on ARAC. Cellular uptake of PD-L1 bound ARAC by endocytosis also lowers the level of membrane PD-L1 expression, allowing the cancer cells to be attacked by cytotoxic T cells. Our study provides evidence for the relationship between PLK1 inhibition and cancer immunosuppression, and supports the combination of PLK1 inhibitor and PD-L1 immune checkpoint blockade as a potential therapeutic option. Further, our study highlights a rational combination strategy to augment existing therapies without increasing toxicity by utilizing a nanoparticle platform as a delivery vehicle.

## Results

### PLK1 inhibition upregulates PD-L1 expression

**Fig. 1:**
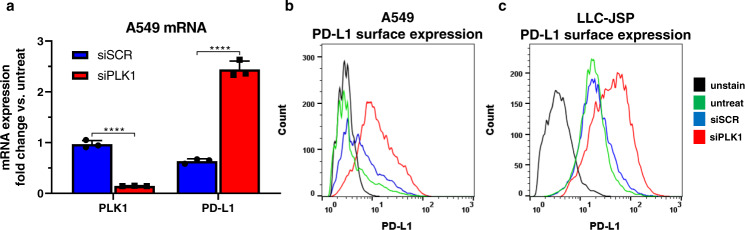
PLK1 knockdown by siRNA induces PD-L1 expression. **a** PLK1 and PD-L1 mRNA expression in A549 (human NSCLC) at 48 h post treatment with PLK1 siRNA (siPLK1) or scrambled siRNA (siSCR) normalized to HPRT housekeeping gene. Dharmafect 1 transfection agent was used. Data presented as mean ± SD from 3 independent samples; ****P < 0.0001 (Unpaired *t*-test; two-tailed). PD-L1 surface expression of (**b**) A549 and (**c**) LLC-JSP at 72 h post treatments assessed by flow cytometry. Data presented as histogram of individual events (cells); ****P < 0.0001 for A549 siPLK1 vs. untreat; ****P < 0.0001 for LLC-JSP siPLK1 vs. untreat (Unpaired *t*-test; two-tailed). Source data are provided as a Source Data file.

We and others have previously reported that PLK1 inhibition or knockdown results in a cell cycle arrest in G2/M phase leading to cancer cell death^14,19–22^. Herein, we find that PLK1 knockdown also increases PD-L1 surface expression in both human (A549) and murine (LLC-JSP) lung cancer cell lines. As shown in Fig. 1a, 85% knockdown of PLK1 mRNA (by siRNA against PLK1) resulted in a 2.5-fold increase in PD-L1 mRNA expression in A549 cell line compared to untreated cells at 2 days after siRNA treatment. This was then confirmed at the surface protein level in A549 (Fig. 1b) and LLC-JSP (Fig. 1c) lung cancer cell lines at 3 days after siRNA treatments.

### Mitotic kinase inhibitor (MKI)-induced PD-L1 expression

Following on the finding that PLK1 inhibition results in PD-L1 upregulation, we sought to determine whether this holds true for inhibition of other mitotic kinases. We screened mitotic kinase small molecule inhibitors against PLK1 (volasertib), Aurora kinase A (alisertib), and CHK1 inhibitor (AZD7762) in human and mouse lung cancer cell lines. As shown in Supplementary Fig. 1, treatment of A549 and LLC-JSP cells with volasertib, alisertib, or AZD7762 resulted in reduced cancer cell viability in a dose-dependent manner. Volasertib and alisertib also caused a significant upregulation of PD-L1 surface expression in both A549 and LLC-JSP cells, while AZD7762 increased surface PD-L1 in A549 cells. Furthermore, in support of our results using PLK1 siRNA (Fig. 1) or volasertib (Supplementary Fig. 1), onvansertib (a newer PLK1 small molecule inhibitor) also reduced cell viability and upregulated PD-L1 expression in A549 cells (Supplementary Fig. 2). Additionally, we confirmed the finding of prior studies^23,24^ that volasertib causes apoptotic cell death (assessed by caspase 3/7 activity) in NSCLC cells (Supplementary Fig. 3). These results establish the link between mitotic kinase inhibition and PD-L1 upregulation, and demonstrate the potency of volasertib over other mitotic kinase inhibitors.

### PLK1 inhibition leads to PD-L1 upregulation through activation of the mitogen-activated protein kinase (MAPK) pathway

**Fig. 2:**
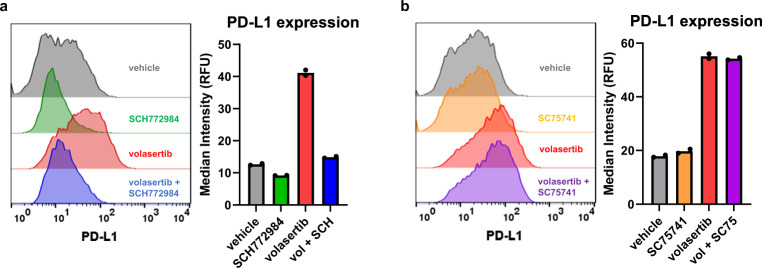
Volasertib-induced PD-L1 upregulation is dependent on MAPK. **a** PD-L1 surface expression of NSCLC cells (H460) treated with vehicle control (0.1% DMSO in PBS), SCH772984 (ERK1/2 small molecule inhibitor, 1 μM), volasertib (100 nM), or SCH772984 (SCH; 1 μM) + volasertib (vol; 100 nM) – (left) representative histograms, (right) MFI quantification. Data presented as mean MFI (median fluorescent Intensity) from biological duplicates, 10,000 events collected per sample. **b** PD-L1 surface expression of H460 cells treated with vehicle control (0.1% DMSO in PBS), SC75741 (NF-kB inhibitor, 1 μM), volasertib (100 nM), or SC75741 (SC75; 1 μM) + volasertib (vol; 100 nM) – (left) representative histograms, (right) MFI quantification. Data presented as mean MFI from biological duplicates, 10,000 events collected per sample. Source data are provided as a Source Data file.

Previous studies have demonstrated the involvement of the MAPK pathway and NF-kB in PD-L1 upregulation after treatment with paclitaxel (mitotic inhibitor chemotherapy)^25,26^. Specifically, Gong et al. reported that paclitaxel-induced PD-L1 expression was MAPK-dependent in colorectal and liver cancers^25^, while Peng et al. found that paclitaxel induces PD-L1 upregulation via NF-kB in ovarian cancer^26^. To decipher the mechanism of PLK1 inhibition-induced PD-L1 expression, we probed downstream signaling pathways after PLK1 inhibition with volasertib. We found that volasertib treatment led to activation of the MAPK pathway, evidenced by an increase in phosphorylated ERK1/2, and, separately, an increase in phosphorylated NF-kB expression (Supplementary Fig. 4). In NSCLC, Jong et al. reported that the MAPK pathway plays a key role in PD-L1 regulation through increasing transcriptional activity and stabilizing PD-L1 (*CD274*) mRNA^27^. Thus, we hypothesized that volasertib induces PD-L1 expression through the MAPK pathway, which is a known regulator of PD-L1 expression^28^. To investigate this, NSCLC cells (H460) were treated with volasertib, ERK1/2 small molecule inhibitor SCH772984, or both drugs, and PD-L1 expression was assessed by flow cytometry. As shown in Fig. 2a, inhibition of ERK1/2 could negate the elevated PD-L1 expression induced by volasertib; cells treated with both volasertib and SCH772984 showed PD-L1 expression level similar to vehicle control-treated cells. The same experiment was then conducted with an NF-kB inhibitor (SC75741) and PD-L1 expression was unaltered (Fig. 2b), suggesting that PD-L1 upregulation is not dependent on NF-kB in NSCLC cells. Similarly in H1437 and H1944 NSCLC cell lines, ERK1/2 inhibitor abrogated the volasertib-induced PD-L1 upregulation, while NF-kB inhibition had no effect (Supplementary Fig. 5).

### Combination of PLK1 inhibition and PD-L1 blockade reduces tumor growth and prolongs survival in mice

**Fig. 3:**
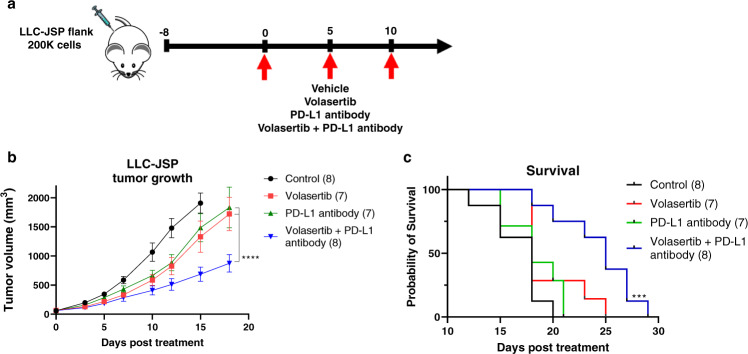
PLK1 inhibition potentiates PD-L1 blockade in syngeneic lung tumors. **a** C57BL/6 mice were injected with 200,000 LLC-JSP cells on the right flank. On day 0 (8 post tumor inoculation), mice were grouped (*n* = 7–8) and received i.p. treatments of control vehicles (PBS and HCl/saline), PLK1 inhibitor volasertib (20 mg/kg), PD-L1 antibody (10 mg/kg), or combination of PLK1 inhibitor and PD-L1 antibody at the same dose. Treatments were administered every 5 days for 3 doses. **b** Tumor growth of mice. Data presented as mean ± SEM; ****P < 0.0001 (mixed model Two-Way repeated measures ANOVA with Tukey’s correction for multiple comparisons). **c** Kaplan–Meier Survival curve. ***P = 0.0007 vs. saline (Log-rank Mantel–Cox test). Source data are provided as a Source Data file.

Based on our finding that PLK1 inhibition upregulates PD-L1 expression, we investigated whether volasertib and a PD-L1 monoclonal antibody would synergize in vivo. We used LLC-JSP cells to develop a flank tumor model in immune-competent mice similar to a previous report^29^. Mice with tumors (>60 mm^3^) were treated i.p. with volasertib, PD-L1 antibody, or the combination of volasertib and PD-L1 antibody as shown in Fig. 3a. The combination treatment (volasertib + PD-L1 antibody) significantly reduced tumor growth compared to each monotherapy and the vehicle-treated mice (Fig. 3b). Additionally, the combination treatment significantly prolonged survival while neither monotherapy (volasertib or PD-L1 antibody) had a statistically significant survival effect compared to the vehicle-treated mice (Fig. 3c).

### PD-L1 antibody conjugated nanoparticles for delivery of PLK1 inhibitor volasertib (ARAC)

**Fig. 4:**
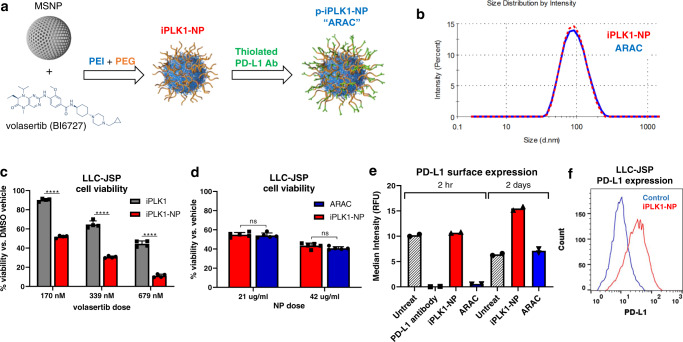
PD-L1 antibody conjugated NP for delivering PLK1 inhibitor volasertib (p-iPLK1-NP; ARAC). **a** Synthesis scheme. Nanoparticle images are re-printed with permission from Wiley^18^; © 2022 Wiley-VCH GmbH. **b** Hydrodynamic size by dynamic light scattering (DLS). **c** Cell viability by CTG assay of LLC-JSP cells treated with volasertib (iPLK1) or iPLK1-NP; all having equivalent volasertib dose as specified. Data presented as mean ± SD from 4 independent samples; ****P < 0.0001 (Unpaired *t*-test; two-tailed). **d** Cell viability by CTG assay of LLC-JSP cells treated with iPLK1-NP or ARAC (p-iPLK1-NP) at equivalent NP and volasertib dose. Data presented as mean ± SD from 5 independent samples; ns – not significant (Unpaired *t*-test; two-tailed). (**e**, **f**) PD-L1 surface expression of the cells treated with PBS, PD-L1 antibody (50 µg/ml), iPLK1-NP (with 210 ng/ml volasertib), or ARAC (with 210 ng/ml volasertib and 1.68 µg/ml antibody) for 2 h and 2 days. Data presented as mean MFI from biological duplicates, 10,000 events collected per sample. Source data are provided as a Source Data file.

Our data show the benefit of combining PD-L1 antibody and PLK1 inhibitor to enhance therapeutic impact. However, each drug alone carries significant toxicity risks that may limit the clinical translation of this combination. To overcome this limitation, we investigated a targeted delivery approach utilizing our polymer modified mesoporous silica nanoparticle (NP) platform which has been proven effective for targeted delivery of siRNA to breast tumors and lung tumors in our prior work^14,16,17,30^. Volasertib (iPLK1) was loaded onto mesoporous silica nanoparticle (MSNP) core prior to surface modification with polyethylenimine (PEI), polyethylene glycol (PEG), and PD-L1 antibody (Fig. 4a). The final composition (ARAC or p-iPLK1-NP) contained 14.3% PEI and 10% PEG (Supplementary Fig. 6c), with 0.5% volasertib and 4.0% PD-L1 antibody (by weight of MSNP). The PEI and PEG compositions were needed to prevent nanoparticles from aggregation before and after antibody loading^31^. Volasertib-loaded nanoparticles, with PD-L1 antibody (ARAC) and without (iPLK1-NP), had a hydrodynamic size of 90 nm (Fig. 4b), and rigid core size of 50 nm (Supplementary Fig. 6a). In agreement with our prior studies, we found that the nanoconstruct can be stored stably at −80 ^o^C and retains the efficacy and hydrodynamic size of fresh material (Supplementary Fig. 6b). Regarding drug release, we found that volasertib was preferentially released from the nanoconstruct in endo/lysosomal solution (pH 4.5) over PBS (pH 7.4) (Supplementary Fig. 6d). As shown in Supplementary Fig. 7, ARAC significantly reduced viability of human (A549 and H460) and mouse (LLC-JSP) lung cancer cells in a dose-dependent manner, while the bare nanoparticle (NP) showed minimal toxicity. Moreover, treatment of LLC-JSP cells with iPLK1-NP significantly reduced cell viability more than free volasertib at the same dose (Fig. 4c). Similar results were obtained with B16-F10 melanoma cells and 4T1 breast cancer cells (Supplementary Fig. 8) suggesting cross-cancer efficacy. In vitro, PD-L1 antibody had no role in cancer killing (see ARAC vs. iPLK1-NP; Fig. 4d) or enhancing the delivery (as all nanoparticles were taken up by cells within 3 days regardless of PD-L1 antibody loading). In agreement with previous findings using PLK1 siRNA (Fig. 1) or free inhibitors, treatment with iPLK1-NP resulted in a significant increase in surface PD-L1 expression on surviving cells at 2 days, but not at 2 hrs post administration (Fig. 4e) since PLK1 inhibition effects (i.e., cell cycle arrest) had not yet transpired. However, nanoparticle-bound PD-L1 antibody was as effective as a 30-fold greater dose of free PD-L1 antibody at reducing surface staining of PD-L1 (see ARAC at 2 h, Fig. 4e), which may be attributed to blockade by the therapeutic antibody or internalization of PD-L1-complexed nanoparticles (Supplementary Fig. 9). This is due to the high concentration of antibodies on the nanoparticles (approximately 2 × 10^3^ antibodies per particle) that the cells encountered. These densely decorated nanoparticles can bind multiple PD-L1 ligands at once and be endocytosed with PD-L1 (i.e., receptor-meditated endocytosis)^32^. Furthermore, Fig. 4f suggests that upon treatment with iPLK1-NP, PD-L1 upregulation in the surviving population is not via selection process (e.g., death of cells with low PD-L1 first) – since control cells (PBS) did not have any high PD-L1 population – but rather is due to signaling effects of PLK1 inhibition. Indeed, PLK1 inhibition also led to upregulation of PD-L1 expression in melanoma and breast cancer cells with varying PD-L1 baseline expression (Supplementary Fig. 8).

### Uptake, feedforward delivery, and specificity of ARAC nanoconstruct

To study the uptake of the nanoconstruct, NSCLC cells were treated with PD-L1 antibody-conjugated nanoparticles (p-NP) carrying a fluorescent dye (Dy677) and incubated at 4 ^o^C for 1 h or 37 ^o^C for 1–4 h prior to imaging. As shown in Supplementary Fig. 9, most p-NP was effectively internalized by NSCLC cells (H460) upon 1 h incubation at 37 ^o^C, while most p-NP stayed on the cell membrane without any significant cellular uptake upon 1 h incubation at 4 ^o^C. This suggests that active endocytosis, as opposed to passive uptake^33^, is the primary uptake mechanism of p-NP. Further, higher PD-L1 staining is observed intracellularly upon 1 h incubation at 37 ^o^C compared to 1 h incubation at 4 ^o^C, suggesting that PD-L1 is internalized with the nanoparticles. Moreover, since most p-NPs are intracellular at 1 h, the observed lower surface PD-L1 expression reported in Fig. 4e (at 2 h time-point) is primarily due to PD-L1 internalization and not due to the nanoparticles’ blocking the PD-L1 staining antibody on the cell membrane.

While ARAC initially engages PD-L1 upon binding and internalization (as shown in Fig. 4e 2 hr time-point and Supplementary Fig. 9), surviving cells have upregulated PD-L1 due to the signaling effects of PLK1 inhibition. In turn, upregulated PD-L1 is used as the homing target for subsequent ARAC, leading to cancer targeting in a feedforward manner (i.e., greater targeting with increased doses of the treatment). To investigate the feedforward targeting of ARAC, we used 4T1 murine cancer cells which express low baseline PD-L1 levels (the lowest of any cancer cell line tested in our study). ARAC led to the upregulation of PD-L1 in 4T1 cells 4 days post treatment (Supplementary Fig. 10a). We then assessed the cellular uptake of ARAC in control 4T1 cells (with low PD-L1) and ARAC-treated 4T1 cells (with upregulated PD-L1). As shown in Supplementary Fig. 10b, after 1 h exposure, ARAC was preferentially taken up by the PD-L1 high cells vs. PD-L1 low cells by nearly 4-fold, demonstrating the selectivity and feedforward targeting by ARAC. We also evaluated the cell killing selectivity by comparing viability of murine cancer cells (LLC-JSP, 4T1, B16-F10) vs. bone marrow-derived dendritic cells (BMDCs). As shown in Supplementary Fig. 10c, treatment led to significant cell killing in cancer cells but minimal killing in dendritic cells. Similar to Fig. 4d, PD-L1 antibody had no effect on enhancing the delivery in this setting since all nanoparticles were taken up by cells within 3 days regardless of PD-L1 expression. Thus, the treatment's selectivity against cancer cells over BMDCs is due to cancer dependence on PLK1, as previously reported^34^.

### ARAC induces anti-tumor immune response in a bilateral lung cancer tumor model

**Fig. 5:**
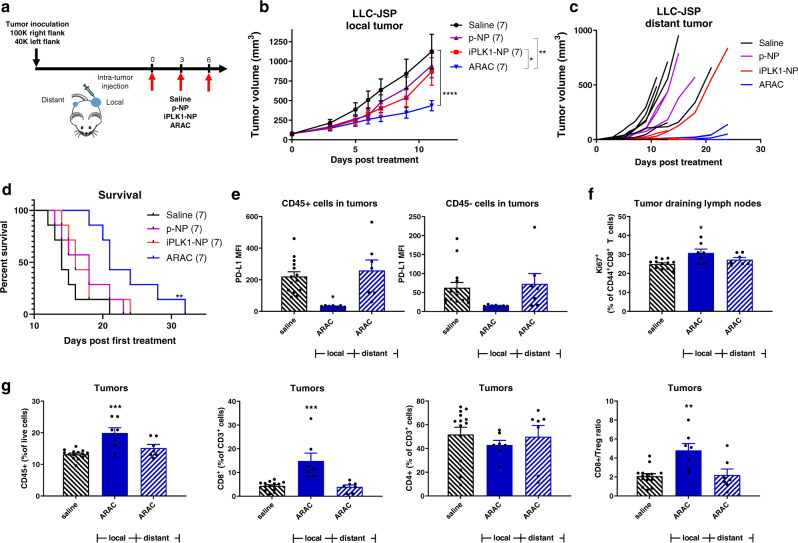
ARAC elicits anti-tumor immune effects. **a** 100,000 LLC-JSP cells were injected in right flank and 40,000 cells were injected in left flank of C57BL/6 mice. On day 12 post tumor inoculation, mice (*n* = 7 per treatment group) received intratumoral treatments of saline, p-NP, iPLK1-NP, or ARAC to the right (local) tumor. Each dose consists of 0.5 mg NP (containing 2.5 µg volasertib and/or 20 µg PD-L1 antibody) in 50 µl per dose for 3 doses total. **b** Local (treated) tumor growth. Data presented as mean ± SEM; *P = 0.0104, **P = 0.0017, ****P < 0.0001 (Two-Way repeated measures ANOVA with Tukey’s correction for multiple comparisons). **c** Distant (untreated) tumor growth of individual mice (distant tumors developed in 6/7 saline, 3/7 p-NP, 3/7 iPLK1-NP, and 2/7 ARAC at shown time-points). **d** Kaplan–Meier Survival curve (mice were euthanized when a combined tumor size reached 2000 mm^3^). **P = 0.0036 for ARAC vs. saline (Log-rank Mantel–Cox test). **e**–**g** Mice (*n* = 7) were inoculated with 250,000 and 100,000 LLC-JSP cells for bilateral tumors and treated as shown in (**a**) with ARAC or saline. One day post 3rd injection, tumors were harvested and processed into single cell suspensions for flow cytometry analysis. **e** PD-L1 expression (median fluorescent intensity; MFI) in CD45+ and CD45− cells. **f** Proliferative effector T cells (%Ki67 of CD44+ CD8+ cells) in tumor-draining lymph nodes. **g** CD45+ (% of live cells), CD8+ (% of CD45+ CD3+ cells), CD4+ (% of CD45+ CD3+ cells), and CD8+/Treg (Regulatory T cells; CD4+ FoxP3+ (% of CD45+ CD3+ cells)) in tumors. Data presented as mean ± SEM; *P < 0.05, **P < 0.01, ***P < 0.001 (One-Way ANOVA with Tukey’s correction for multiple comparisons). Source data are provided as a Source Data file.

To assess the anti-tumor immune effect of ARAC, we utilized a bilateral flank tumor model. C57BL/6 mice were injected with 100,000 and 40,000 LLC-JSP cells on the right and left flank, respectively. At day 12 post inoculation, the mice were grouped (*n* = 7) and the right flank (local) tumors were injected with PBS, p-NP (NP with PD-L1 antibody), iPLK1-NP (NP with volasertib), or ARAC as shown in Fig. 5a. Growth of local (treated) and distant (untreated) tumors was monitored. Treatments with ARAC significantly reduced the growth of local tumors when compared to p-NP or iPLK1-NP (Fig. 5b). We did not observe significant tumor reduction with iPLK1-NP since the volasertib administered in this study (0.125 mg/kg intratumoral for 3 doses) was much lower than the efficacious dose range reported for volasertib in xenograft tumors (e.g., 10–40 mg/kg once or twice a week systemically)^35–37^. Similarly, the PD-L1 antibody dose on p-NP (20 μg for 3 doses) is lower than the efficacious dose (200 μg i.p. once a week) reported in this model. As shown in Fig. 5c, a delay in the onset of distant tumors was also observed in ARAC group, suggesting that a systemic anti-tumor immune response was generated. Further, ARAC significantly prolonged survival of mice when compared to saline or single drug NPs (Fig. 5d). In a separate study, mice were inoculated with 250,000 and 100,000 LLC-JSP cells for bilateral tumors and treated as shown in Fig. 5a with ARAC. Tumors and tumor-draining lymph nodes were harvested one day after the final treatment for immune profiling by flow cytometry. As shown in Fig. 5e, PD-L1 surface expression was reduced in ARAC-treated tumors in both hematopoietic (CD45+ , primarily immune cells) and nonhematopoietic (CD45−, primarily cancer cells) cell populations, confirming the efficacy of PD-L1 antibody on the nanoconstruct. ARAC also significantly enhanced proliferation (Ki67+) of effector CD8+ T cells in the local tumor-draining lymph nodes (Fig. 5f). Longer time-points (than one day post dosing) may be needed to see significant changes in distant tumors to decipher the observed delayed onset of the distant tumors in Fig. 5c. Importantly, ARAC-treated tumors had a significantly higher population of total immune cells (CD45+ ) and CD8+ T cells, and a significantly higher CD8+/Treg ratio compared to control tumors (Fig. 5g).

### NP delivery reduces effective doses of PLK1 inhibitor and PD-L1 antibody in lung tumor mice by 5-fold

**Fig. 6:**
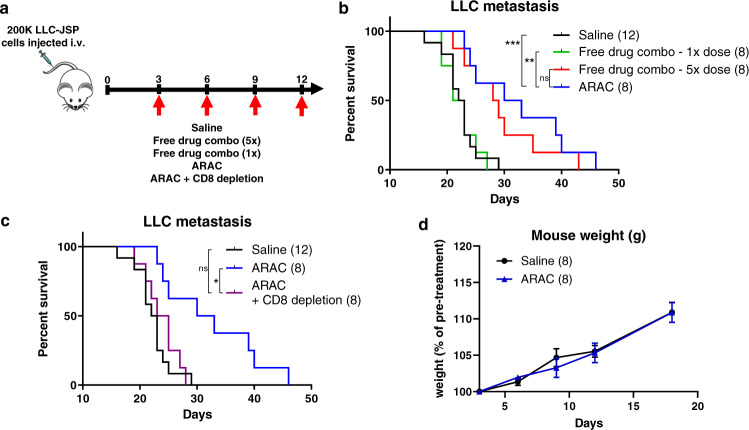
ARAC improves survival of mice bearing metastatic lung tumors. **a** C57BL/6 mice were injected with 200,000 LLC-JSP cells intravenously. After 3 days, mice were treated with saline, free drugs (i.p., at 1x dose: 2.5 µg volasertib and 20 µg PD-L1 and 5x dose: 12.5 µg volasertib and 100 µg PD-L1 antibody), ARAC (i.v., containing 2.5 µg volasertib and 20 µg PD-L1), or ARAC + anti-CD8 (200 μg i.p. twice weekly). Treatments were administered every 3 days for a total of 4 doses. **b**, **c** Kaplan–Meier Survival curve. *P = 0.0120, **P = 0.0033, ***P = 0.0009, ns – not significant (Log-rank Mantel–Cox test). **d** Mouse weight change post first treatment; data presented as mean ± SEM. Source data are provided as a Source Data file.

To evaluate ARAC systemically, we developed an experimental metastatic lung tumor model by intravenous (i.v.) injection of LLC-JSP cells (200,000 cells), which developed tumors mainly in the lungs (confirmed at sacrifice). Mice were randomly grouped and treated i.v. via tail vein with saline, free drugs (volasertib + PD-L1 antibody at the same dose as or 5-fold higher than the ARAC dose), ARAC, or ARAC plus anti-CD8 antibody (Fig. 6a). Mice treated with ARAC survived significantly longer than those treated with saline or free drugs at the same dose (***p < 0.001 vs. saline; **p < 0.01 vs. free drugs (1x)) (Fig. 6b) and slightly better than those treated with the 5-fold dose of the free drug combo (*p < 0.05 vs. saline). Thus, delivery with ARAC could effectively reduce required dose of drug by at least 5-fold. Moreover, ARAC’s efficacy was confirmed to be immune-mediated, as CD8+ T cell depletion by anti-CD8 antibodies abolished the prolonged survival of ARAC-treated mice (Fig. 6c). Furthermore, treatment with ARAC did not cause any weight loss, demonstrating its favorable safety profile in mice (Fig. 6d).

### ARAC efficacy in ICI-refractory KLN-205 syngeneic tumor model

**Fig. 7:**
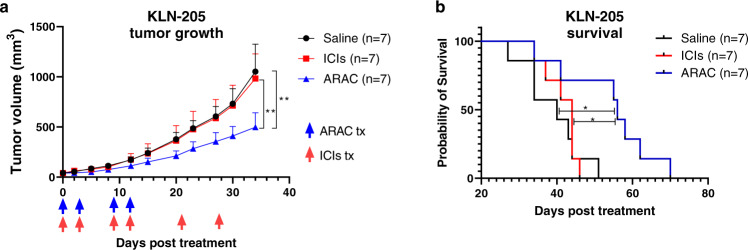
ARAC reduces tumor growth and prolongs survival of mice bearing KLN-205 murine lung tumors. **a** DBA/2 mice were injected with 500,000 KLN-205 cells in 100 μL PBS on the right flank. 13 days post inoculation, mice were grouped (*n* = 7) and treated with saline, ICIs (PD-1 and CTLA-4 antibodies – i.p. 200 μg/dose and 100 μg/dose respectively – for 6 doses), or ARAC (i.v. 50 mg/kg for 4 doses). **a** KLN-205 tumor growth; blue arrows specify ARAC dosing days, red arrows specify ICI dosing days. Data presented as mean + SEM; **P = 0.0011 for ARAC vs. saline, **P = 0.0041 for ARAC vs. ICIs (mixed model Two-Way repeated measures ANOVA with Tukey’s correction for multiple comparisons). **b** Kaplan–Meier Survival curve. *P = 0.0101 for ARAC vs. saline, *P = 0.0231 for ARAC vs. ICIs (Log-rank Mantel–Cox test). Source data are provided as a Source Data file.

We further evaluated ARAC’s in vivo efficacy in an ICI-refractory tumor model, KLN-205^38,39^. We compared ARAC efficacy to dual ICI treatments of PD-1 and CTLA-4 antibodies (which was recently granted FDA approval for metastatic NSCLC). DBA/2 mice were inoculated with 500,000 KLN-205 cells on the right flank. When tumors developed (~40 mm^3^), mice were grouped (*n* = 7) and treated i.v. with 50 mg/kg ARAC (for 4 doses on days 0, 3, 9, 12), i.p. with ICIs (PD-1 and CTLA-4 antibodies – 200 μg/dose and 100 μg/dose respectively – for 6 doses on days 0, 3, 9, 12, 21, 28), or saline. As shown in Fig. 7a, ARAC treatments significantly reduced tumor growth when compared to saline (**p < 0.01) and ICIs (**p < 0.01), while ICIs had no effect on tumor growth when compared to saline. Moreover, mice treated with ARAC survived significantly longer than saline or ICI-treated mice (Fig. 7b).

## Discussion

The potential of PLK1 inhibition as a therapeutic strategy has been well studied in various cancer types; however, the interplay of PLK1 inhibition with cancer immunity remains mostly unexplored. In this study, we report that PLK1 inhibition results in increased PD-L1 expression in cancer cells. In NSCLC, PLK1 inhibition-induced PD-L1 upregulation is driven by the MAPK pathway, as inhibition of ERK1/2 abolished the observed PD-L1 upregulation by volasertib. The upregulation of PD-L1 suggests that evasion of the immune response is one of the mechanisms exploited by cancer cells that survive PLK1 inhibition. Consequently, we show that the combination of PLK1 inhibitor and PD-L1 antibody significantly reduces tumor progression in mice compared to each drug alone.

To overcome the dose limiting toxicity of current PLK1 inhibitors that prevents them from advancing beyond clinical trials, we developed a PLK1 inhibitor-loaded nanoparticle and conjugated it to PD-L1 antibody (ARAC) to synergize the effects of PLK1 inhibition and PD-L1 blockade. In our prior works, we reported on the efficacy and safety profile of this nanoparticle platform in delivering siRNA to mediate gene knockdown of breast and lung tumors in vivo^14,17^. The sol gel MSNP synthesis and layer-by-layer modification on MSNPs offer good reproducibility and scalability. We have successfully scaled up the synthesis protocol to yield 16 g of nanoparticles (2.5-L reaction volume), which is over 100-fold higher than our small-scale synthesis^40^. We have also reported excellent batch-to-batch reproducibility of the nanoconstruct synthesis for chemical and physical properties and bioactivities^14,16^. Regarding stability, we found that the nanoparticles are stable for at least 4 months at −20 °C (longer time is being investigated) and for years in terms of size and efficacy at −80 °C^41^. We have also shown that our antibody-conjugated nanoparticles can be lyophilized without losing their physical characteristics and efficacy over time^40^. Furthermore, to date, our NP platform has exceeded required safety criteria, demonstrating low cytotoxicity in multiple organ cell lines (<10% cell death)^30^; great blood and blood immune cell (PBMC) compatibility^16^; excellent safety after 7 doses given systemically to mice over 1 month (no adverse effects on body weight, serum biomarkers, and histology of kidney and liver)^18^; good maximum tolerated dose (MTD not reached at 4-fold of efficacious dose)^18^; and effective clearance as MSNP is soluble to benign silicic acid^42,43^ at serum pH and cleared in urine^41,44^.

In this study, we demonstrate that the nanoparticle platform can improve delivery of small molecule inhibitors (i.e., volasertib) as treatment with PLK1 inhibitor on nanoparticles reduced cell viability significantly more than free PLK1 inhibitor. We also demonstrate that PLK1 inhibitor and PD-L1 antibody synergize in vivo when co-delivered as free drugs and as an NP construct (ARAC). In mice bearing bilateral tumors, 3 doses of ARAC administered intratumorally reduced the growth of local (injected) tumors significantly more than nanoparticle delivering a single drug and delayed the onset of distant tumors suggesting that a systemic anti-tumor response was triggered. Immune profiling of tumors showed that ARAC could effectively reduce PD-L1 expression in both immune (CD45+ ) and cancer (CD45−) cells. The effect of ARAC on PD-L1 expression is dynamic; it first reduces PD-L1 (upon NP-mediated ligand engagement and internalization), then later increases PD-L1 in the surviving cells due to inhibition of PLK1 by volasertib, until receiving a subsequent dose. Further, ARAC significantly increased the number of CD8+ tumor infiltrating lymphocytes and increased the CD8+/Tregs ratio, indicating that the treatment could reshape the tumor microenvironment to an immune-permissive state. Additionally, the therapeutic benefit of nanoparticle delivery was demonstrated in an experimental metastatic lung tumor model, where i.v. administration of ARAC improved survival as much as a 5-fold higher dose of the free drugs. This suggests that nanoparticle delivery can overcome dose limiting toxicity issues of PLK1 inhibitors and thereby facilitate clinical utility. ARAC’s efficacy was confirmed to be immune-mediated as CD8 depletion abolished the prolonged survival. Importantly, in an ICI-refractory tumor model, ARAC retained effectiveness and significantly reduced tumor growth and prolonged survival of mice, which suggests that ARAC may overcome the clinical limitation of immune checkpoint inhibitors. Presently, although the combination of PLK1 inhibition and immune checkpoint blockade improved overall survival in mouse models – a benefit to patients if recapitulated in the clinic – it failed to induce tumor regressions. Since ARAC’s effect is mainly mediated by CD8+ T cells, strategies to improve adaptive anti-tumor immunity and T cell activity, such as enhancing antigen presentation with an immune adjuvant or administering immune checkpoint inhibitors in combination, will be evaluated in our subsequent work.

**Fig. 8:**
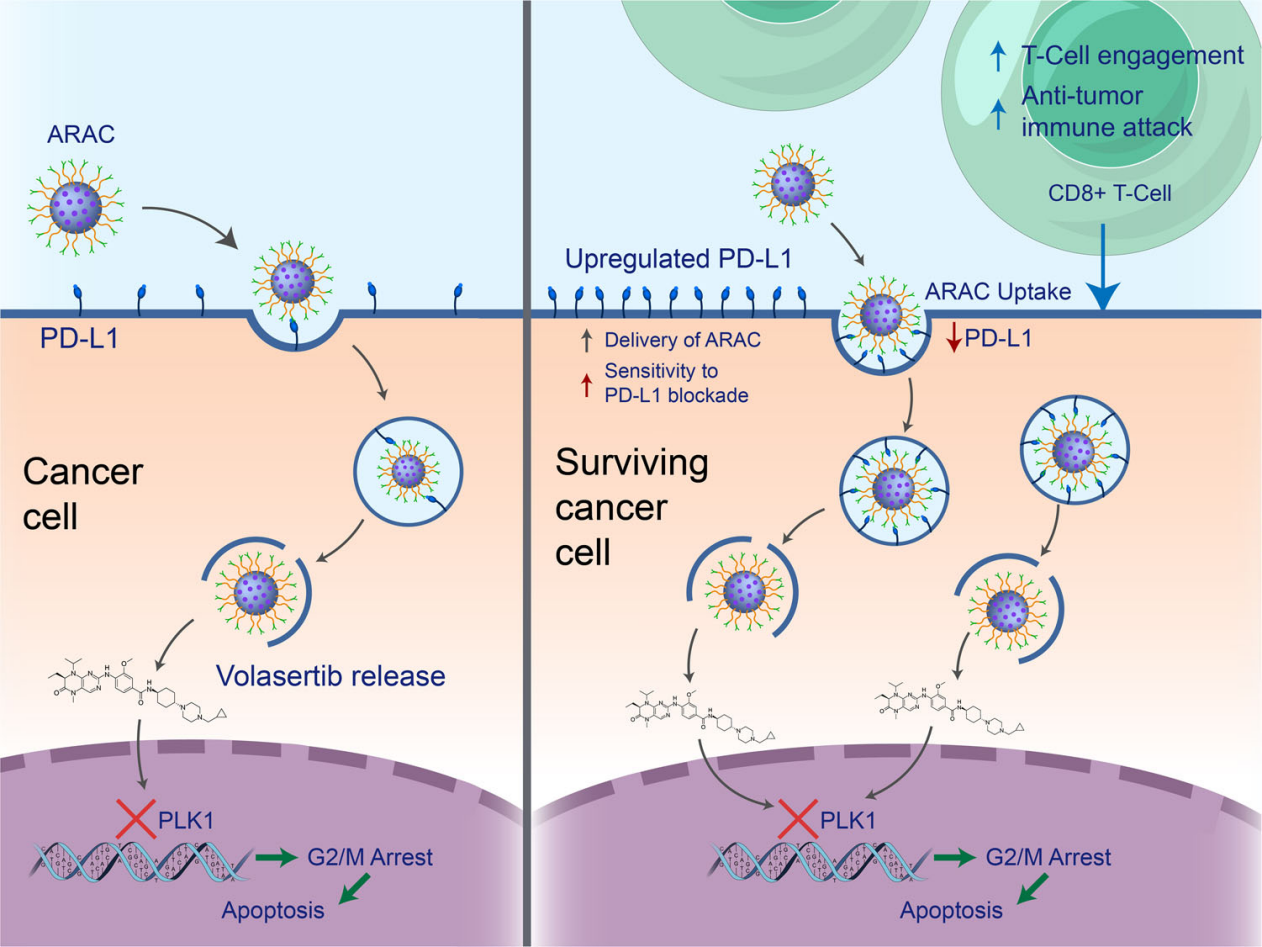
Proposed mechanism of action of ARAC nanoconstruct. (Left cell) ARAC binds to PD-L1 on cancer cell surface and is internalized via receptor-mediated endocytosis. Endosomal escape of ARAC is mediated by PEI polymer^16^ and volasertib is released to inhibit PLK1 activity, leading to G2/M cell cycle arrest and apoptotic cell death. However, G2/M arrest induced by volasertib upregulates PD-L1 levels in surviving cancer cells, thereby rendering them unresponsive to immune-mediated effects (due to PD-L1-mediated immunosuppression). (Right cell) We capitalize on this property by utilizing elevated PD-L1 levels in surviving cancer cells as the homing target for subsequent ARAC delivery, leading to cancer targeting in a feedforward manner (i.e., higher targeting with increased doses of the treatment). Enhanced delivery of ARAC results in loss of PD-L1, which allows for cytotoxic CD8+ T cells to effectively kill cancer and generate an anti-tumor immune response.

Mechanistically, ARAC leads to cell cycle arrest and generation of an anti-tumor immune response, while exhibiting a unique feedforward delivery capability (i.e., greater delivery to surviving cancer cells having upregulated PD-L1 levels from an initial treatment) to mount an anti-tumor immune attack (Fig. 8). For tumors with initially low PD-L1 levels, ARAC, which has a slight positive charge (6 mV in 10 mM NaCl), may rely first on the enhanced permeability and retention effect (EPR) and adsorptive endocytosis. Subsequently, it would rely on PD-L1 antibody mediated endocytosis after volasertib-induced PD-L1 upregulation in the surviving cancer cells. While toxic to multiple cancer cells, ARAC is safe to antigen-presenting cells (DCs) needed for priming anti-tumor T cells.

Our research herein focused on lung cancer, the deadliest cancer^45^. Like melanoma, in which immunotherapy has been most effective, lung cancer is a disease with a high mutational load – an attribute that drives the expression of various neo-epitopes that can be recognized by the host immune system^46,47^. Consequently, immunotherapy is a promising approach to treating lung cancer. However, objective response rates are much lower for lung cancer patients than melanoma. The research described here illustrates how superior responses can be achieved for lung cancers by combining PLK1 inhibition with PD-L1 blockade. Further, cytotoxic agents other than PLK1 inhibitor have also been shown to increase PD-L1 expression, including paclitaxel in ovarian cancer^26^, CDK4/6 inhibitors^48^, and PARP inhibitors^49^ in breast cancer. Therefore, it is logical that these drugs are now in clinical investigations in combination with PD-1/PD-L1 checkpoint blockade^50^. Our findings suggest that co-delivering these drugs and PD-L1 antibody with our nanoparticle construct will increase efficacy while lowering toxicity. Further, as PLK1 is overexpressed in a variety of cancers, the combination of PLK1 inhibition and PD-L1 blockade may have broad application. Finally, other mitotic kinase inhibitors that elevate PD-L1 expression of cancer cells should also be combined with PD-L1 immune checkpoint blockade to improve treatment outcomes in clinics.

## Methods

All animal studies were reviewed and approved by Institutional Animal Care and Use Committee (IACUC) at Oregon Health and Science University (OHSU).

### Cell lines and reagents

A549 (CCL-185), H460 (HTB-177), H1437 (CRL-5872), and H1944 (CRL-5907) NSCLC cells were purchased from ATCC and maintained in RPMI media with 10% fetal bovine serum (FBS). Mouse cancer cell lines KLN205 (CRL-1453), B16F10 (CRL-6475), and 4T1 (CRL-2539) were purchased from ATCC and maintained in EMEM + 10% FBS, DMEM + 10% FBS, and RPMI + 10% FBS, respectively. Lewis Lung Carcinoma (LLC) metastatic variant, LLC-JSP cells were gift from Dr. Don Gibbons lab (MD Anderson Cancer Center), and were cultured in RPMI + 10% FBS. BMDCs were harvested from naïve C57BL/6 mice and cultured following published protocols^51,52^. Staining antibodies used: human PD-L1 (PE; clone MIH1, Biolegend #393608, dilution 1:20), mouse PD-L1 (PE; clone MIH5, BD Biosciences #558091, dilution 1:20), mouse CD8 (BV650; clone 53-6.7, BD Biosciences #563234, dilution 1:200), mouse CD4 (BV711; clone RM4-4, BD Biosciences #740651, dilution 1:200), mouse CD45 (APC-Cy7; clone 30-F11, Biolegend #103116, dilution 1:400), mouse CD3 (PerCP5.5; clone 17A2 Biolegend #100218, dilution 1:20), mouse/human CD44 (FITC; clone IM-7 Biolegend #103022, dilution 1:400), mouse Ki-67 (efluor450; clone SolA15, invitrogen #48-5698-82, dilution 1:20), mouse FoxP3 (Alexa-647; clone MF-14, Biolegend #126408, dilution 1:200), human PD-L1 antibody (unconjugated; clone MIH1, eBioscience #14-5983-82, dilution 1:50). Alexa Fluor 488 secondary antibody was purchased from Life Technologies (#A11001, dilution 1:1000). In vivo grade (*InVivo*Mab) anti-mouse PD-L1 antibody (BE0101; clone 10 F.9G2), anti-mouse PD-1 antibody (BE0146; RMP1-14), anti-mouse CTLA-4 antibody (BE0131; clone 9H10), and anti-mouse CD8α antibody (BE0061; clone 2.43) were purchased from BioXcell. Pharmaceutical-grade human PD-L1 antibody avelumab (Pfizer/Merck KGaA) was purchased from OHSU Pharmacy. Small molecule inhibitors (volasertib, alisertib, AZD7762, onvansertib, SC75741, SCH772984) were purchased from Selleckchem. All inhibitors were solubilized in DMSO at high concentrations and diluted down to 1% DMSO (in PBS pH 7.2) for working concentrations – final DMSO content in wells (exposed to cells) was 0.1% DMSO. SiRNA sequences: PLK1 (antisense 5’-UAUUCAUUCUUCUUGAUCCGG-3’); scrambled SCR (antisense 5’-UUAGUCGACAUGUAAACCA-3’) and Dharmafect 1 Transfection Reagent were purchased from Dharmacon (Horizon).

### Nanoparticle synthesis and characterization

Bare MSNPs were synthesized as we have previously reported^16^. For PLK1 inhibitor loading, volasertib was mixed with MSNPs in ethanol for overnight shaking at room temperature (350 RPM). The next day, nanoparticles were coated with 10 kDa branched PEI (Alfa Aesar) and 5 kDa mal-PEG-NHS (Jenkem) following our previous studies^16,40^. For PD-L1 antibody conjugation, in vivo grade mouse PD-L1 antibody (BioXcell) or avelumab was buffer exchanged to PBS pH 8 (Zeba spin column, Thermo Fisher) and thiolated using Pierce Traut’s reagent (Thermo Fisher) following manufacturer’s protocol. Thiolated antibody was added to NP at 20 wt.% and shaken overnight at 4 ^o^C (300 RPM). Nanoparticles were washed with PBS pH 7.2 before characterization. To determine polymer loading and final nanoparticle concentration, 1 mg nanoparticles were heated to 900 ^o^C (20 ^o^C/min) using TGA Q50 (TA Instruments). Nanoparticle size was determined using Zetasizer (Malvern, ZS-90). Antibody loading was determined by protein quantification of NP supernatant with Pierce BCA protein assay (Thermo Fisher). To quantify PLK1 inhibitor loading, nanoparticles were shaken in DMSO solution (75% DMSO, 25% PBS) for 24 h to release the drug and supernatant was collected. Absorbance (330 nm) of supernatant was measured with Infinite 200 Pro plate reader (Tecan) to determine loading extent. For drug release study over time, nanoparticles were incubated in PBS pH 7.4 (1X, Gibco) or lysosomal simulant solution pH 4.5 (100 mM NaCl, 100 mM KCl, 100 mM sodium acetate; acidified to pH 4.5 with 1 M HCl) at a final NP concentration of 4 mg/mL. Samples were shaken on a temperature controlled orbital shaker at 100 RPM, 37 °C. At indicated time points, aliquots were removed and centrifuged to pellet the nanoparticles and supernatant was collected to quantify volasertib released by UV-vis (absorbance at 330 nm). For Transmission electron microscopy (TEM), 5 μL of nanoparticle preparations were deposited onto glow discharged (60 s 15 mAmp, negative mode) carbon formvar 400 Mesh copper grids (Ted Pella 01822-F) for 3 min, rinsed 15 s in water, wicked on Whatman filter paper 1, stained for 3 min in filtered 1% (w/v) uranyl acetate in water, wicked and air dried. Samples were imaged at 120 kV on a FEI Tecnai^TM^ Spirit TEM system. Images were acquired using the AMT software interface on a Nanosprint12S-B cMOS camera system.

ARAC refers to nanoparticle loaded with both PLK1 inhibitor (volasertib) and PD-L1 antibody, p-NP is nanoparticle loaded with PD-L1 antibody, and iPLK1-NP is nanoparticle loaded with PLK1 inhibitor. For the majority of studies (in mice or using murine cell lines), in vivo grade mouse PD-L1 antibody (clone 10 F.9G2; BioXCell) was used. For human cell lines, avelumab (Pfizer/Merck kGAa) was used as the PD-L1 antibody on NP.

### Flow cytometry

For in vitro studies, cells were plated in 6-well plates and treated with indicated treatments. Three days post treatments, cells were collected and washed in FACS buffer prior to staining. Primary (unconjugated or fluorophore-conjugated) and secondary antibodies were stained for 30 min and 1 h, respectively, under rocking on ice. After staining, cells were washed in FACS buffer before analysis with Guava easyCyte (Millipore Sigma) flow cytometer (10,000 events per sample). For immune profiling, tumors and tumor-draining lymph nodes were harvested and cut into small sections for digestion. Tissues were digested in digestion media (1 mg mL^−1^ Collagenase D and 0.1 mg mL^−1^ DNase I in HBSS) at 37 °C for 30 min and mechanically dissociated by passing through 70 µm pore nylon cell strainers. Red blood cells in the sample were lysed by incubating in RBC lysis buffer (Alfa Aesar) at room temperature for 5 min. Cells were washed twice with PBS and stained with Live/Dead Fixable Aqua Stain (Thermo Fisher Scientific) for 15 min. Cells were washed twice with FACS buffer (1% BSA in PBS), incubated with FcR blocking solution (Tonbo Biosciences) for 5 min, and then stained for a select panel of surface-staining antibodies for 15 min at room temperature. Intracellular staining (for FoxP3 and Ki67) was performed with BD Cytofix/Cytoperm (BD Biosciences), following the manufacturer’s protocol after cell surface staining. Samples were washed twice with FACS buffer and resuspended in FACS buffer for analysis. All data were acquired with a BD LSRFortessa flow cytometer (OHSU’s Flow Cytometry Core), and analyzed using FlowJo Software (TreeStar Inc.). Only live cells (determined by live–dead stain occurring before fixing/permeabilization) were analyzed.

### Cell viability and apoptosis post treatments

Cells (1500–2000/well) were plated in white flat bottom 96 well plate overnight. The following day, cells were treated with small molecule inhibitors or drug loaded nanoparticles and controls as indicated and media was changed 24 h post treatment. 2–3 days post treatment, cell viability was assessed using Cell Titer Glo assay (Promega) following manufacturer’s instructions. Apoptosis (assessed 2 days post treatment) was quantified using Caspase-Glo 3/7 assay system (Promega). Luminescence was read with Tecan plate reader.

### RT-qPCR to assess PLK1 and PD-L1 mRNA

Cells were seeded in 6-well plates and treated with PBS, siSCR, or siPLK1 using Dharmafect 1 Transfection Agent (Horizon), and media was changed after 24 h. Two days post transfection, RNA was isolated with GeneJet RNA purification kit (Thermo Fisher Scientific) following manufacturer’s instructions. One-Step qRT-PCR was performed using EXPRESS One-Step Superscript™ qRT-PCR Kit (Invitrogen). Cycling conditions: 50 ^o^C for 2 min, 95 ^o^C for 10 min, 40 cycles of 95 ^o^C for 15 s, and 60 ^o^C for 1 min. TAQMAN gene expression primers Human *HPRT* mRNA (Hs99999909_m1), Human *PLK1* mRNA (Hs00983225_g1), and Human PDL1 (Hs00204257_m1) were used. Data was analyzed using 2^–ΔΔC(t)^ method.

### Western blot

NSCLC cells were seeded in 100 mm dishes (300,000 cells/dish) overnight and treated with indicated treatments. Cell culture medium was changed one day after treatment. Three days post treatment, cells were lysed in RIPA buffer. Lysate was sonicated and centrifuged (21,130 x *g* for 15 min) and supernatant was collected. Amount of total protein was quantified using BCA. 30 μg of proteins (per sample) were mixed with 4X Novex NuPAGE LDS sample buffer and beta-mercaptoethanol (10% final concentration). Samples were denatured for 5 min at 95 ^o^C and loaded onto gel (NuPAGE) for electrophoresis. Proteins were then transferred onto PVDF-FL membrane and blocked with LI-COR Intercept (TBS) blocking buffer. Membranes were incubated with primary antibodies overnight (Phospho-NF-kB p65 (Ser536) (93H1) antibody #3033, dilution 1:1000; Phospo-p44/42 MAPK (ERK1/2) (Thr202/Tyr204) antibody #9101, dilution 1:1000; β-Actin (8H10D10) antibody #3700, dilution 1:1000) at 4 ^o^C. Next day, membranes were rinsed with TBS-T and IRDye conjugated secondary antibodies (IRDye 680RD Goat anti-Rabbit (LI-COR; 926-68071) and IRDye 800CW Goat anti-Mouse (LI-COR; 926-32210); or IRDye 800CW Donkey anti-Rabbit (LI-COR; 926-32213) and IRDye 680RD Goat anti-Mouse (LI-COR; 926-68070)) were added (dilutions 1:10,000) for 1 hour under rocking at room temperature. Membranes were scanned on a LI-COR Odyssey imaging system.

### Syngeneic tumor models and treatments

For single tumor LLC-JSP model (Fig. 2), LLC-JSP murine lung cancer cells (200,000) were inoculated in right flank of NCI C57BL/6NCr female mice (6 weeks) (Charles River NCI colony; strain code 556). At 8 days post tumor inoculation, mice received intraperitoneal (i.p.) treatments of volasertib (20 mg/kg) and/or PD-L1 antibody (10 mg/kg) every 5 days for 3 doses total. For bilateral LLC-JSP tumors, C57BL/6NCr female mice (6 weeks) were inoculated with 100,000 and 40,000 LLC-JSP cells in right and left flank, respectively. At 12 days post inoculation, the aforementioned treatments were administered intratumorally to the right tumor every 3 days for 3 doses total. For KLN205 tumors, DBA/2 female mice (6 weeks) (Charles River NCI colony; strain code 026) were inoculated in right flank with 500,000 KLN205 cells. Starting at 13 days post inoculation, mice received treatments of saline (i.v.), ARAC (i.v.), or PD-1 and CTLA-4 antibodies (i.p.) on indicated days. Tumors were measured with Vernier Caliper and volume calculated by V = 0.5 x length x width^2^. For both single flank and bilateral flank tumor models, mice were sacrificed when total tumor burden exceeded 2000 mm^3^. For metastatic lung tumor model, LLC-JSP (200,000) were injected intravenously (i.v.) to 6 week old C57BL/6NCr female mice. At 3 days post cancer cell injection, mice were randomly grouped and treated with i.v. saline, i.v. ARAC (25 mg/kg NP), i.v. ARAC plus i.p. CD8 antibody (10 mg/kg; twice a week), or i.p. PD-L1 antibody (5 mg/kg or 1 mg/kg) plus volasertib (0.625 mg/kg or 0.125 mg/kg) every 3 days for a total of 4 doses. All studies were reviewed and approved by Institutional Animal Care and Use Committee (IACUC) at Oregon Health and Science University (OHSU). The maximum tumor burden permitted under the IACUC at OHSU was not exceeded in any instances.

### Statistical analysis

GraphPad Prism 8.0 (GraphPad Software Inc.) was used for all statistical analysis. Comparison between two groups was performed with Student’s *t* test. Comparison between more than two groups was performed with One-Way ANOVA with Tukey’s correction for multiple comparisons. Tumor growth was analyzed using two-way repeated measures ANOVA with Tukey’s correction for multiple comparisons. Kaplan–Meier survival curve was analyzed using the log-rank (Mantel–Cox) method. Significance was set at *p* < 0.05. In vitro data are expressed as mean ± SD; in vivo data are expressed as mean ± SEM.

### Reporting summary

Further information on research design is available in the Nature Research Reporting Summary linked to this article.

## Supplementary information

Supplementary Information

Reporting Summary

## Data Availability

The data that support the findings of this study are available from the manuscript and its supplementary information. All source data is provided as a supplementary file with this paper. Source data are provided with this paper.

## Source data

Source Data
