# Supplementary material for: Development of a nanoparticle-based immunotherapy targeting PD-L1 and PLK1 for lung cancer treatment
Source: Nat Commun. 2022 Jul 23;13:4261. doi: 10.1038/s41467-022-31926-9 (PMC9308817; doi:10.1038/s41467-022-31926-9)
Supplement: Supplementary file 1 — Supplementary Information [file 41467_2022_31926_MOESM1_ESM.pdf]

Development of a nanoparticle-based immunotherapy targeting PD-L1 and PLK1 for lung cancer treatment

Moataz Reda[1,2], Worapol Ngamcherdtrakul[1], Molly A. Nelson[1], Natnaree Siriwon[2], Ruijie Wang[1], Husam Y. Zaidan[1], Daniel S. Bejan[1], Sherif Reda[1], Ngoc Ha Hoang[2], Noah A. Crumrine[1], Justin P.C. Rehwaldt[1], Akash Bindal[2], Gordon B. Mills[3,4], Joe W. Gray[2,4], Wassana Yantasee[1,2,4#]

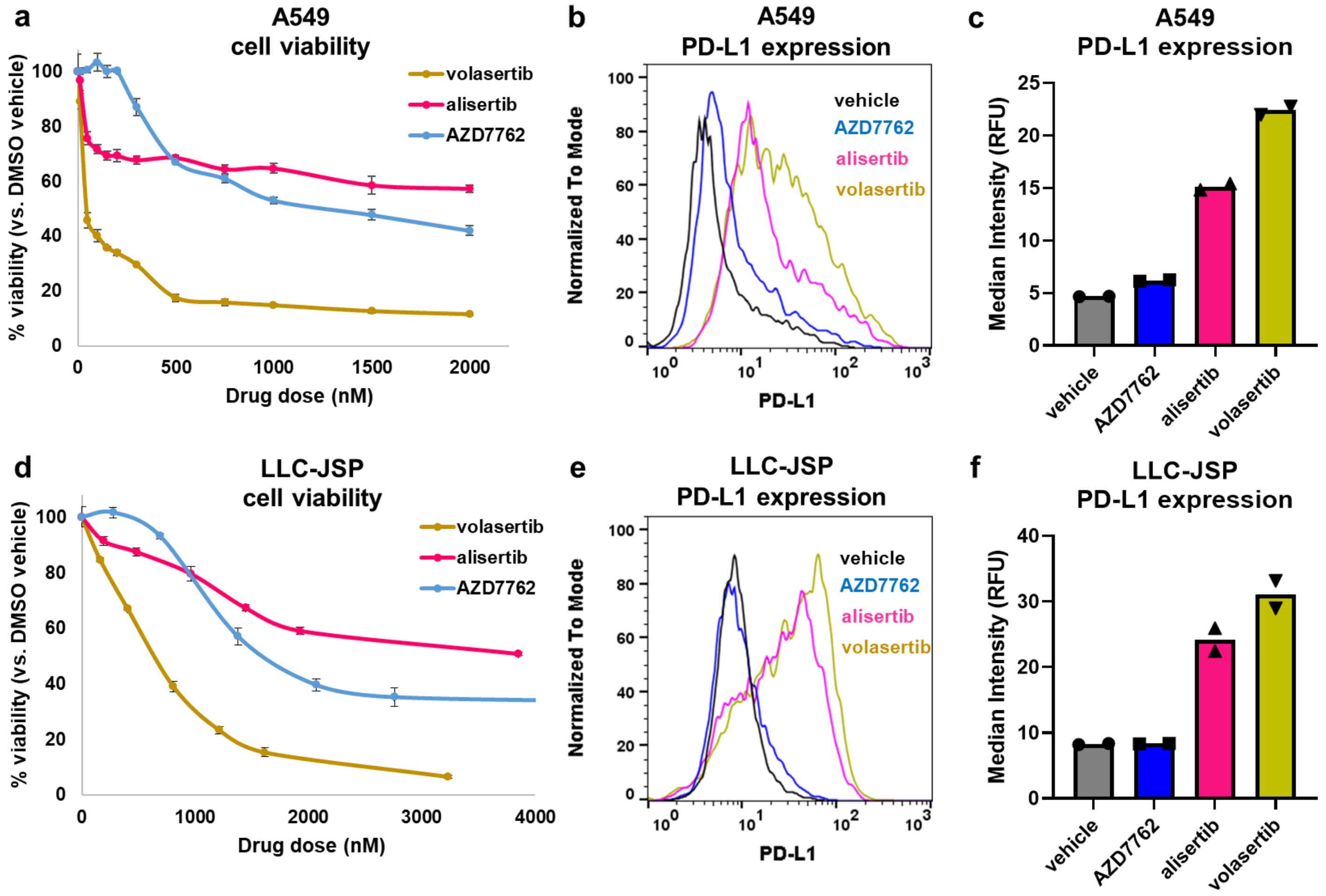

Supplementary Figure 1. Treatment effects of mitotic kinase inhibitors (MKIs). (a) 3-day cell viability dose response of A549 cells treated with volasertib, alisertib, or AZD7762 with indicated doses. Data presented as mean ± SD from 3 independent samples. (b-c) PD-L1 expression levels of A549 cells treated with vehicle control (0.1% DMSO in PBS), AZD7762 (500 nM), alisertib (200 nM), or volasertib (100 nM); (b) Representative histogram and (c) MFI quantification; data presented as mean MFI (median fluorescent Intensity) from biological duplicates, 10,000 events collected per sample. (d) 3-day cell viability dose response of LLC-JSP cells treated with volasertib, alisertib, or AZD7762 with indicated doses. Data presented as mean from 3 independent samples. (e-f) PD-L1 expression levels of LLC-JSP cells treated with vehicle control (0.1% DMSO in PBS), AZD7762 (500 nM), alisertib (200 nM), or volasertib (100 nM); (e) Representative histogram and (f) MFI quantification; data presented as mean MFI from biological duplicates, 10,000 events collected per sample. Source data are provided as a Source Data file.

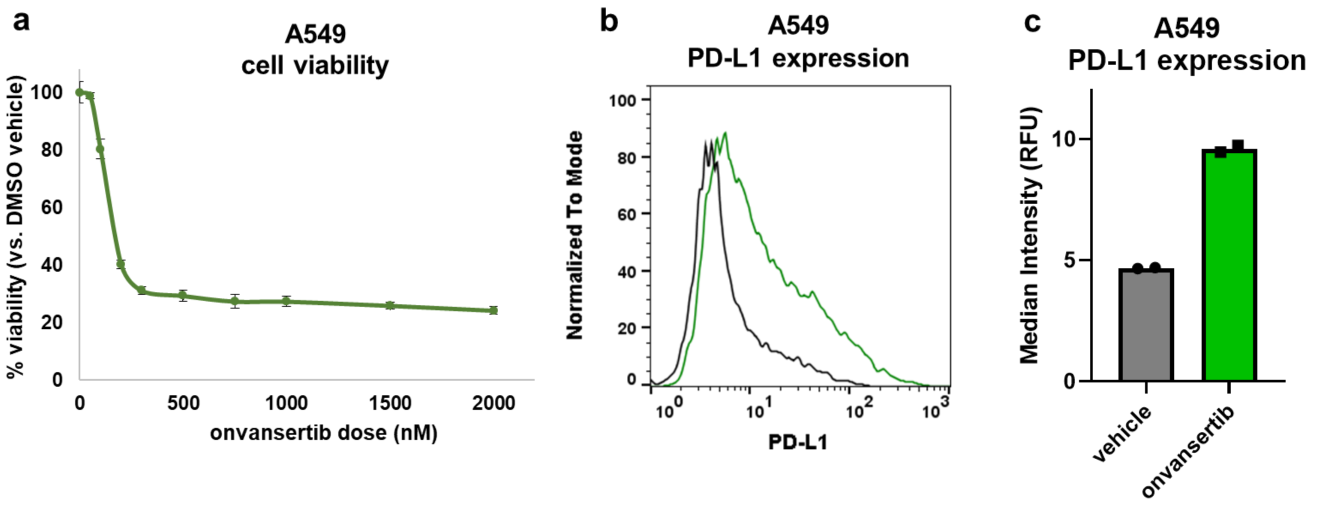

Supplementary Figure 2. Treatment effects of onvansertib (PLK1 inhibitor). (a) 3-day cell viability dose response of A549 cells treated with onvansertib with indicated doses. Data presented as mean ± SD from 4 independent samples. (b-c) PD-L1 expression levels of A549 cells treated with vehicle control (0.1% DMSO in PBS) or onvansertib (100 nM); (b) Representative histogram and (c) MFI quantification; data presented as mean MFI from biological duplicates, 10,000 events collected per sample. Source data are provided as a Source Data file.

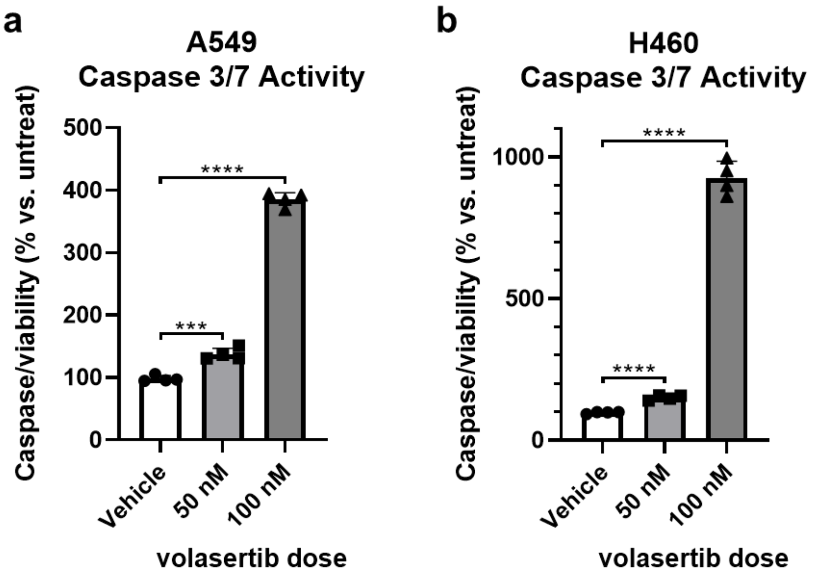

Supplementary Figure 3. Volasertib induces apoptotic cell death in NSCLC cells. Caspase 3/7 activity in (a) A549 and (b) H460 cells treated with vehicle control (0.1% DMSO in PBS), 50 nM volasertib, or 100 nM for 2 days. Caspase activity normalized to cell viability (by CTG) for each sample. Data presented as mean ± SD from 4 independent samples; ***P=0.0003, ****P<0.0001 (Unpaired t-test; two-tailed). Source data are provided as a Source Data file.

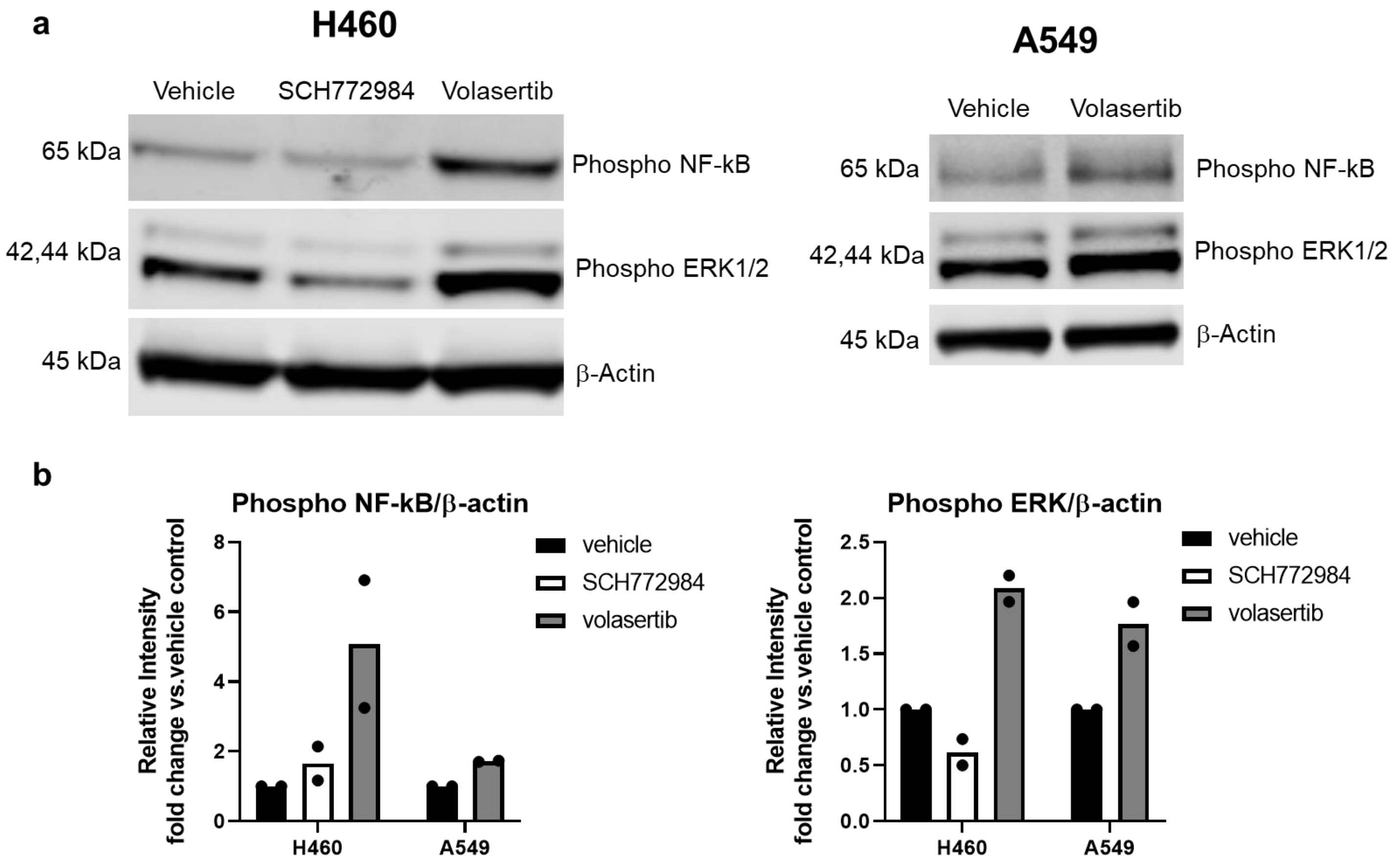

Supplementary Figure 4. NSCLC cells downstream protein expressions. (a) Representative immunoblots of phosphorylated NF-kB p65 (Ser536) (93H1), phosphorylated p44/42 MAPK (ERK1/2) (Thr202/Tyr204), and β-Actin (8H10D10) 3 days post treatment. (Left) H460 NSCLC cells treated with vehicle control (0.1% DMSO in PBS), SCH772984 (1 μM), or volasertib (100 nM). (Right) A549 NSCLC cells treated with vehicle control (0.1% DMSO in PBS) or volasertib (100 nM). (b) Quantification of (left) phosphorylated NF-kB and (right) phosphorylated ERK1/2 normalized by β-Actin loading control. Data presented as mean relative intensity from biological duplicates (fold change vs. vehicle control). Source data are provided as a Source Data file.

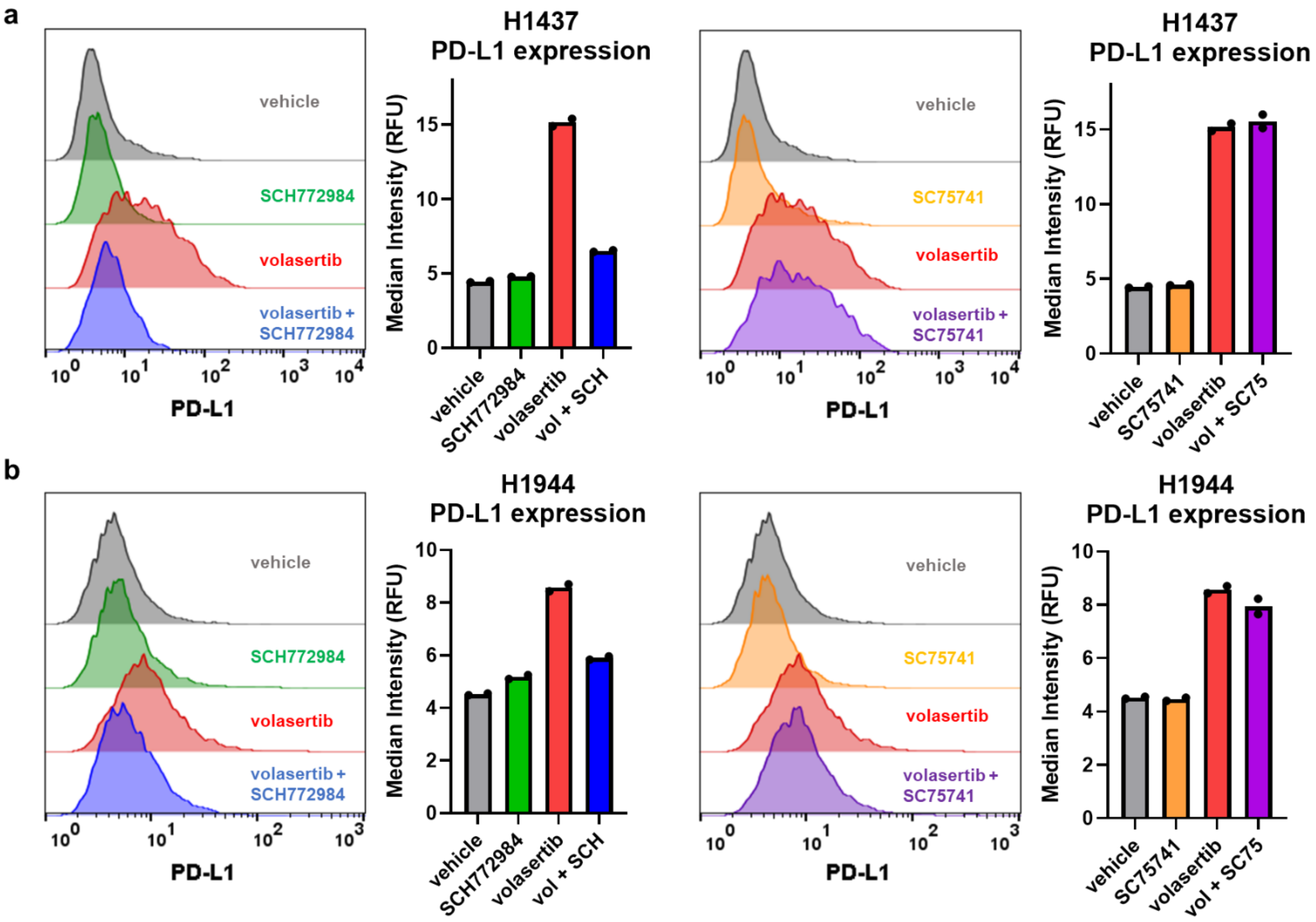

Supplementary Figure 5. Volasertib-induced PD-L1 upregulation is dependent on MAPK. (a) PD-L1 surface expression of H1437 cells treated with vehicle control (0.1% DMSO in PBS), SCH772984 (1 μM), SC75741 (1 μM), volasertib (100 nM), SCH772984 + volasertib, or SC75741 + volasertib – (left) representative histograms, (right) MFI quantification. Data presented as mean MFI from biological duplicates, 10,000 events collected per sample. (b) PD-L1 surface expression of H1944 cells treated with vehicle control (0.1% DMSO in PBS), SCH772984 (1 μM), SC75741 (1 μM), volasertib (100 nM), SCH772984 + volasertib, or SC75741 + volasertib – (left) representative histograms, (right) MFI quantification. Data presented as mean MFI from biological duplicates, 10,000 events collected per sample. Source data are provided as a Source Data file.

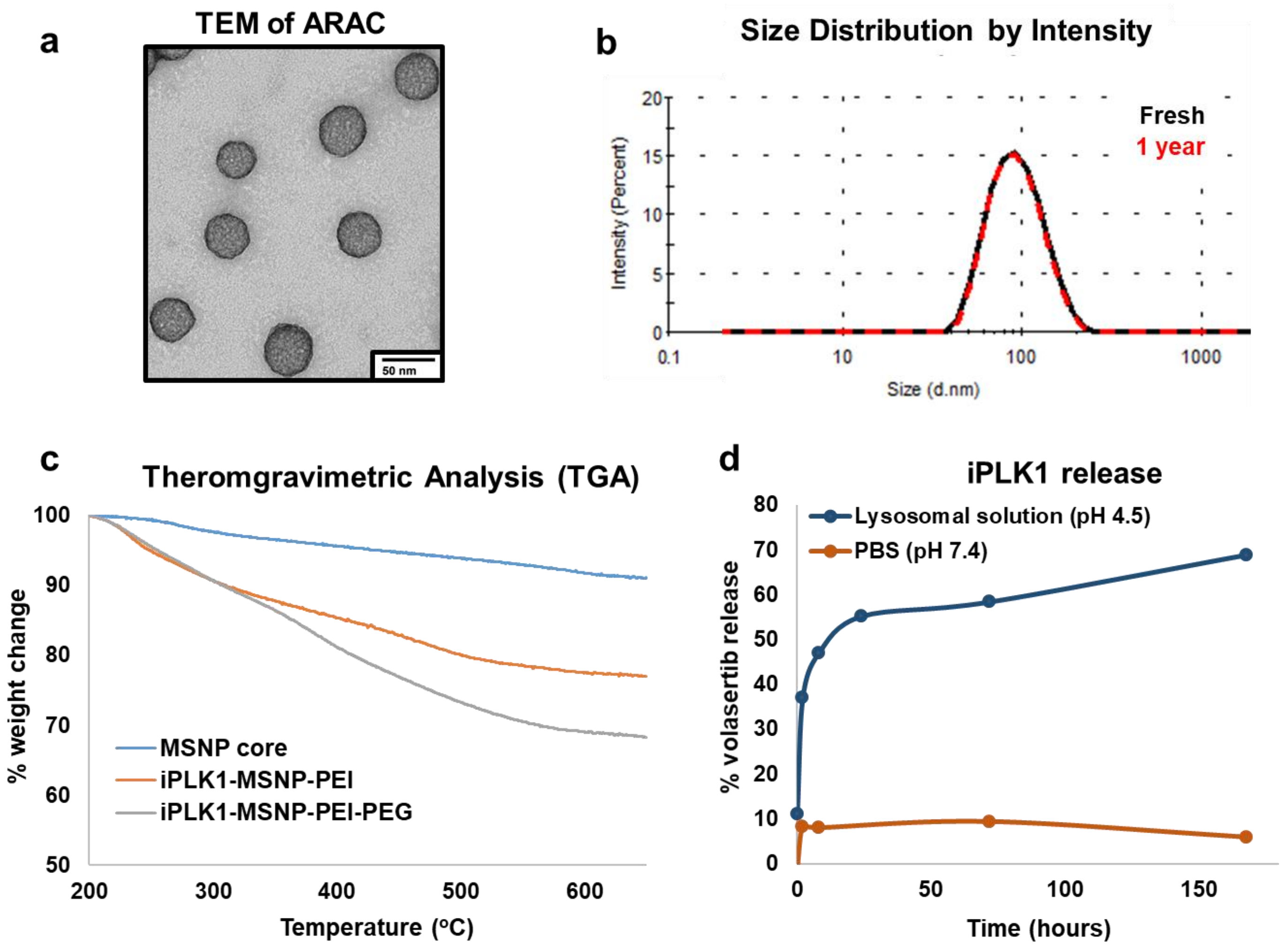

Supplementary Figure 6. Nanoparticle characterization. (a) TEM image of final ARAC construct. (b) Hydrodynamic size of ARAC post-synthesis (fresh) or after 1 year storage at -80°C. (c) Thermogravimetric curves of bare MSNP core, iPLK1-MSNP-PEI, and iPLK1-MSNP-PEI-PEG (ARAC) used to determine polymer loading. (d) iPLK1 (volasertib) release (quantified by UV-vis absorbance at 330 nm) from the nanoconstruct in lysosomal solution (pH 4.5) or PBS (pH 7.4) over time. Data presented as mean (% release) from two independent samples. Source data are provided as a Source Data file.

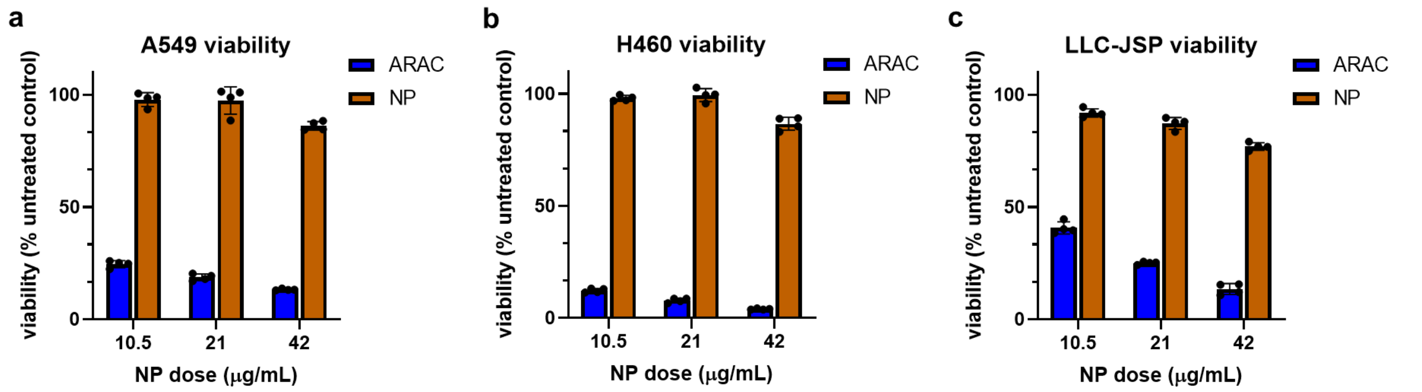

Supplementary Figure 7. ARAC efficacy in NSCLC cells. 3-day cell viability of (a) A549, (b) H460, and (c) LLC-JSP cells treated with ARAC or bare nanoparticle (NP, containing no APIs) at specified doses of nanoparticle. Nanoconstructs were stored for 5 months at -80ºC prior to treatment. Data presented as mean ± SD from 4 independent samples. Source data are provided as a Source Data file.

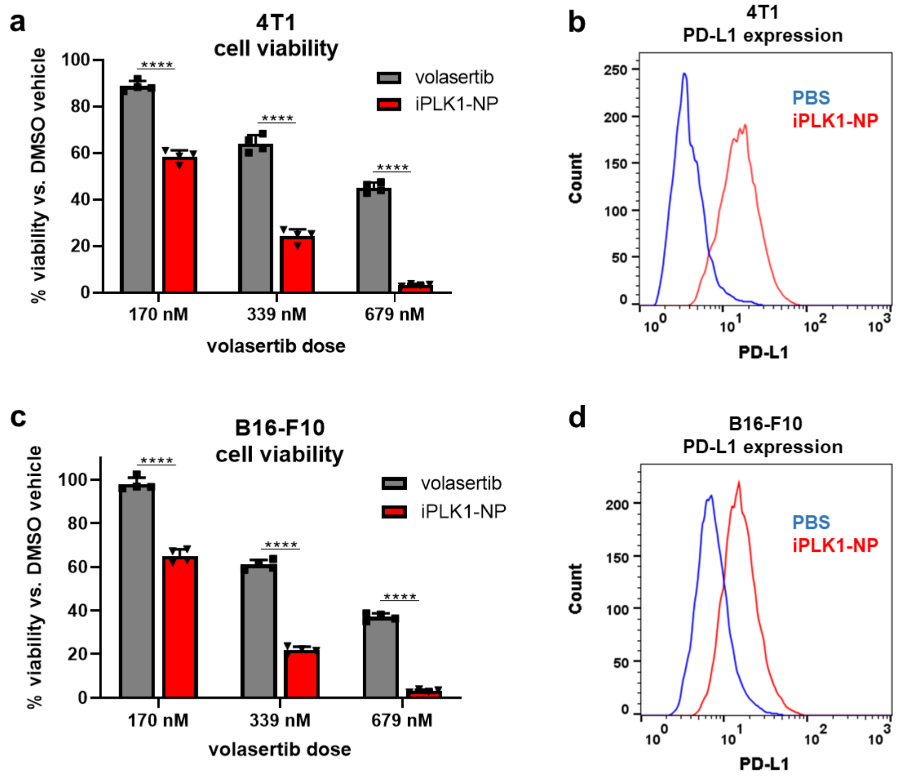

Supplementary Figure 8. Nanoparticle delivery of PLK1 inhibitor volasertib (iPLK1-NP). Viability of (a) 4T1 breast cancer or (c) B16-F10 melanoma cells treated with volasertib (in 1%DMSO/PBS), iPLK1-NP (in PBS), or 1%DMSO/PBS for 3 days. Data presented as mean ± SD from 4 independent samples; ****P<0.0001 (Unpaired t-test; two-tailed). PD-L1 surface expression of (b) 4T1 or (d) B16-F10 cells treated with PBS or iPLK1-NP (42 μg/ml NP, 210 ng/ml volasertib). Source data are provided as a Source Data file.

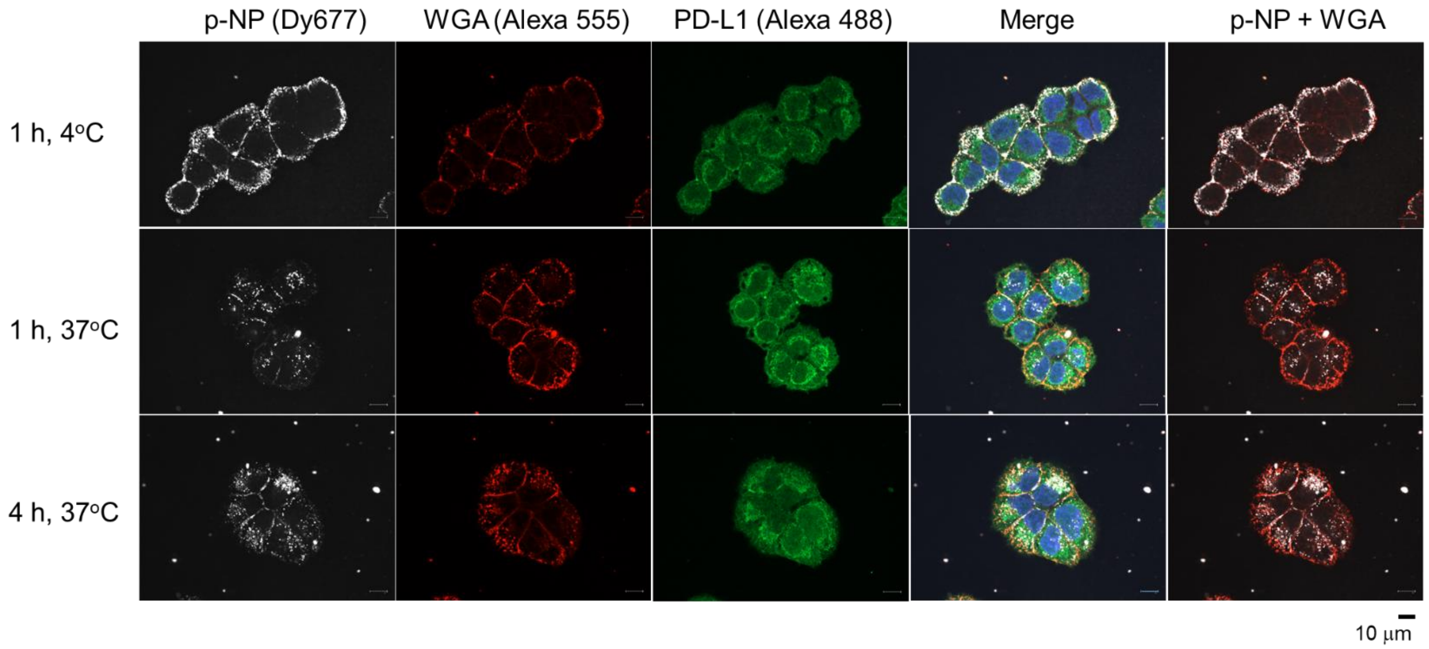

Supplementary Figure 9. PD-L1 antibody-conjugated nanoparticle (p-NP) uptake in NSCLC cells. H460 NSCLC cells were treated with p-NP carrying a fluorescent dye (Dy677) for 1 h at 4°C, 1 h at 37°C, or 4 h at 37°C. Cells were then fixed and stained for WGA (Alexa 555), PD-L1 (Alexa 488), and DAPI prior to imaging with Zeiss CellObserver Spinning Disk confocal microscope, using 63x1.4 NA lens and Hamamatsu Orca Flash 4 v2 camera. Individual channels and merged images are shown for each condition; scale bar = 10 μm. The experiment was independently repeated for each condition (biological duplicates) with similar results; a representative image is shown per condition.

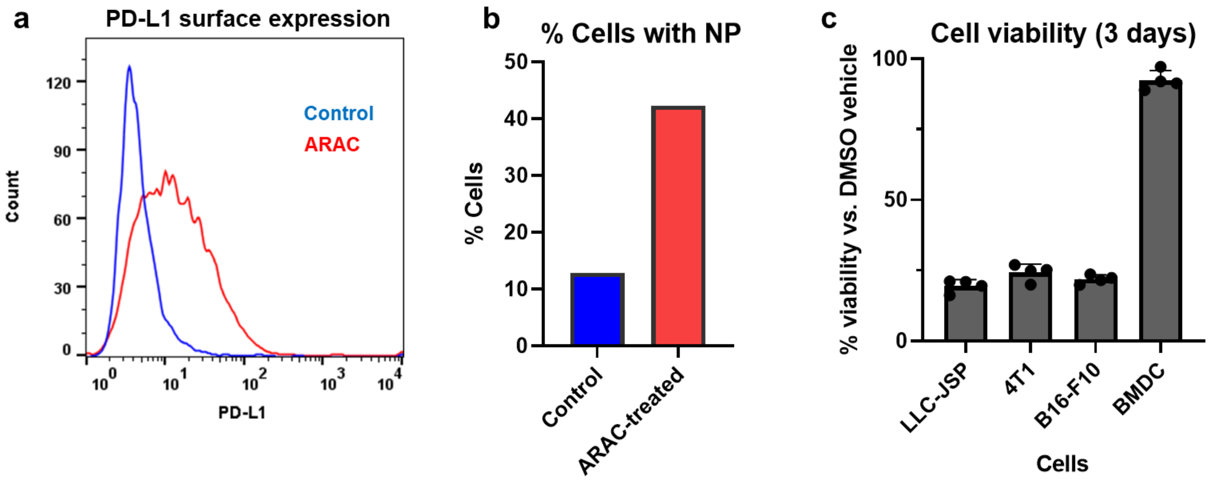

Supplementary Figure 10. Targeting and treatment specificity of ARAC. (A) PD-L1 expression of 4T1 cells 4-day post treatment of ARAC. Cells (PBS-treated (control) and ARAC-treated) were harvested and incubated with ARAC tagged with dye-tagged siRNA for 1 hr. (B) Cellular uptake of ARAC after 1 hr. (C) Cell viability of murine cancer cells (LLC-JSP, 4T1, B16-F10) and murine bone marrow-derived dendritic cells (BMDC) post treatment. Data presented as mean ± SD from 4 independent samples. Source data are provided as a Source Data file.

a

Gating strategies for lymph node

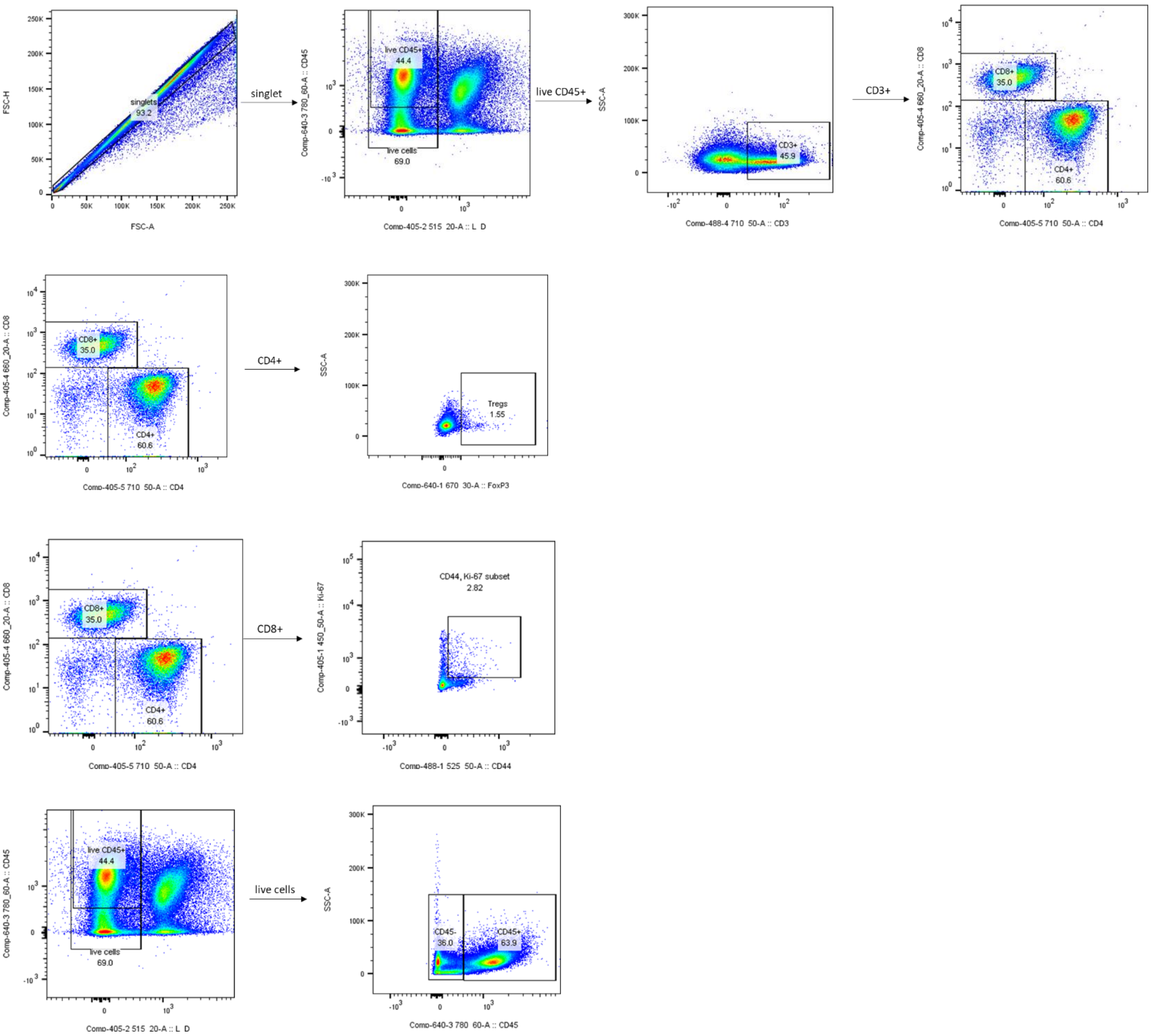

b

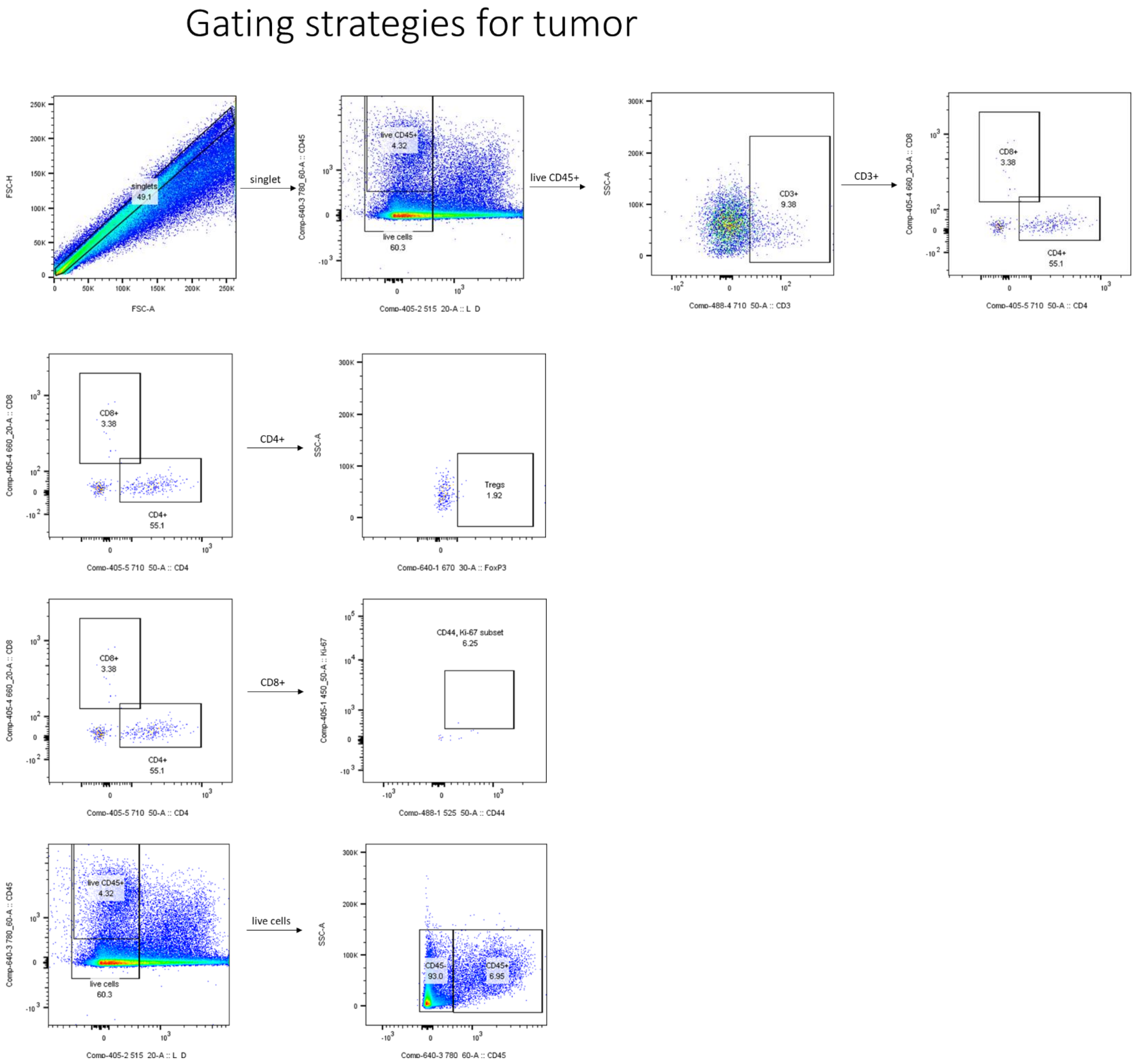

Supplementary Figure 11. Flow cytometry gating strategy for analysis. Gating strategy shown for (a) lymph node samples and (b) tumor samples.
