# Supplementary material for: Development of a nanoparticle-based immunotherapy targeting PD-L1 and PLK1 for lung cancer treatment
Source: Nat Commun. 2022 Jul 23;13:4261. doi: 10.1038/s41467-022-31926-9 (PMC9308817; doi:10.1038/s41467-022-31926-9)
Supplement: Supplementary file 2 — Reporting Summary [file 41467_2022_31926_MOESM2_ESM.pdf]

nature research

Corresponding author(s): Yantasee
Last updated by author(s): Jul 5, 2022

Reporting Summary

Nature Research wishes to improve the reproducibility of the work that we publish. This form provides structure for consistency and transparency in reporting. For further information on Nature Research policies, see our Editorial Policies and the Editorial Policy Checklist.

Statistics

For all statistical analyses, confirm that the following items are present in the figure legend, table legend, main text, or Methods section.

n/a	Confirmed	
☐	☒	The exact sample size (n) for each experimental group/condition, given as a discrete number and unit of measurement
☐	☒	A statement on whether measurements were taken from distinct samples or whether the same sample was measured repeatedly
☐	☒	The statistical test(s) used AND whether they are one- or two-sided<br>*Only common tests should be described solely by name; describe more complex techniques in the Methods section.*
☐	☒	A description of all covariates tested
☐	☒	A description of any assumptions or corrections, such as tests of normality and adjustment for multiple comparisons
☐	☒	A full description of the statistical parameters including central tendency (e.g. means) or other basic estimates (e.g. regression coefficient) AND variation (e.g. standard deviation) or associated estimates of uncertainty (e.g. confidence intervals)
☐	☒	For null hypothesis testing, the test statistic (e.g. F, t, r) with confidence intervals, effect sizes, degrees of freedom and P value noted<br>*Give P values as exact values whenever suitable.*
☒	☐	For Bayesian analysis, information on the choice of priors and Markov chain Monte Carlo settings
☒	☐	For hierarchical and complex designs, identification of the appropriate level for tests and full reporting of outcomes
☒	☐	Estimates of effect sizes (e.g. Cohen's d, Pearson's r), indicating how they were calculated

Our web collection on statistics for biologists contains articles on many of the points above.

Software and code

Policy information about availability of computer code

Data collection	Tecan i-control 2.0<br>Malvern Zetasizer software v7.13<br>TA Thermal Advantage for Q series software Q50<br>Millipore guavaSoft 3.3<br>BD FACSDiva software<br>AMT Imaging software
Data analysis	GraphPad Prism (v.8.0)<br>FlowJo (v.10)<br>Microsoft Excel 2016<br>LI-COR Image Studio Lite (v5.2)

For manuscripts utilizing custom algorithms or software that are central to the research but not yet described in published literature, software must be made available to editors and reviewers. We strongly encourage code deposition in a community repository (e.g. GitHub). See the Nature Research guidelines for submitting code & software for further information.

Data

Policy information about availability of data

All manuscripts must include a data availability statement. This statement should provide the following information, where applicable:

- Accession codes, unique identifiers, or web links for publicly available datasets
- A list of figures that have associated raw data
- A description of any restrictions on data availability

The data that support the findings of this study are available from the manuscript and its supplementary information. All source data is provided as a supplementary file with this paper.

Field-specific reporting

Please select the one below that is the best fit for your research. If you are not sure, read the appropriate sections before making your selection.

☒ Life sciences ☐ Behavioural & social sciences ☐ Ecological, evolutionary & environmental sciences

For a reference copy of the document with all sections, see nature.com/documents/nr-reporting-summary-flat.pdf

Life sciences study design

All studies must disclose on these points even when the disclosure is negative.

Sample size	No statistical methods were used to predetermine sample size for this study. The sample size was determined based on our prior studies investigating the effects of nanoparticle therapeutics on in vitro effects and tumor growth/survival of mice (Ngamcherdtrakul et al. Adv Funct Mater. 2015 May 13; 25(18): 2646–2659; Gu et al. Oncotarget. 2016 Mar 22;7(12):14727-41; Morry et al. Mol Cancer Ther. 2017 Apr;16 (4):763-772; Reda et al. Cancer Lett. 2019 Dec 28;467:9-18)
Data exclusions	No data exclusions.
Replication	Material synthesis and characterization were replicated 5+ times with similar outcomes in each case. All in vitro cell assays included 2-5 independent (biological) replicates. All attempts at replication were successful. For in vivo studies, each treatment group consisted of 6-8 mice/group receiving multiple doses. In vivo studies were not repeated.
Randomization	For in vitro studes, cells were plated in 6-well or 96-well plates at the same cell number using a single stock of cell suspension. The following day, wells were randomly assigned as treatment-wells, control-wells, or untreated-wells. For in vivo studies, mice were grouped by tumor size on first day of treatment, allowing for a similar average starting tumor size for both local and distant tumors across all groups, then each group was randomly assigned treatment.
Blinding	In vitro treatments were not blinded since each experiment was typically conducted/analyzed by a single investigator. For in vivo studies, the treatment was known to investigators who administered the treatment. Downstream assays (i.e. immune-profiling) were done by another investigator who was blinded to the treatment and only received mouse ID numbers during data collection/processing.

Reporting for specific materials, systems and methods

We require information from authors about some types of materials, experimental systems and methods used in many studies. Here, indicate whether each material, system or method listed is relevant to your study. If you are not sure if a list item applies to your research, read the appropriate section before selecting a response.

Materials & experimental systems

n/a	Involved in the study	
☐	☒	Antibodies
☐	☒	Eukaryotic cell lines
☒	☐	Palaeontology and archaeology
☐	☒	Animals and other organisms
☒	☐	Human research participants
☒	☐	Clinical data
☒	☐	Dual use research of concern

Methods

n/a	Involved in the study	
☒	☐	ChIP-seq
☐	☒	Flow cytometry
☒	☐	MRI-based neuroimaging

Antibodies

Antibodies used	Flow cytometry staining antibodies used: human PD-L1 (PE; clone MIH1, Biolegend #393608, dilution 1:20), mouse PD-L1 (PE; clone MIH5, BD Biosciences # #558091, dilution 1:20), mouse CD8 (BV650; clone 53-6.7, BD Biosciences #563234, dilution 1:200), mouse CD4 (BV711; clone RM4-4, BD Biosciences #740651, dilution 1:200), mouse CD45 (APC-Cy7; clone 30-F11, Biolegend #103116,

dilution 1:400), mouse CD3 (PerCP5.5; clone 17A2 Biolegend #100218, dilution 1:20), mouse/human CD44 (FITC; clone IM-7 Biolegend #103022, dilution 1:400), mouse Ki-67 (efluor450; clone SolA15, invitrogen #48-5698-82, dilution 1:20), mouse FoxP3 (Alexa-647; clone MF-14, Biolegend #126408, dilution 1:200), human PD-L1 antibody (unconjugated; clone MIH1, eBioscience #14-5983-82, dilution 1:50). Alexa Fluor 488 secondary antibody was purchased from Life Technologies (#A11001, dilution 1:1000).

Western Blot primary antibodies (1:1000 dilutions): Phospho-NF-kB p65 (Ser536) (93H1) antibody #3033 (Cell Signaling Technologies), Phospo-p44/42 MAPK (ERK1/2) (Thr202/Tyr204) antibody #9101 (Cell signaling Technologies), b-Actin (8H10D10) antibody #3700 (Cell Signaling Technologies). Secondary antibodies (1:10,000 dilutions): IRDye 680RD Goat anti-Rabbit (LI-COR; 926-68071), IRDye 800CW Goat anti-Mouse (LI-COR; 926-32210), IRDye 800CW Donkey anti-Rabbit (LI-COR; 926-32213), IRDye 680RD Goat anti-Mouse (LI-COR; 926-68070).

In vivo grade (InVivoMab) anti-mouse PD-L1 antibody (BE0101; clone 10F.9G2), anti-mouse PD-1 antibody (BE0146; RMP1-14), anti-mouse CTLA-4 antibody (BE0131; clone 9H10), and anti-mouse CD8a antibody (BE0061; clone 2.43) were purchased from BioXcell. Pharmaceutical-grade human PD-L1 antibody avelumab (Pfizer/Merck KGaA) was purchased from OHSU Pharmacy.

Validation

Validation of primary antibodies (from manufacturer) -

Staining antibodies from Biolegend:
-Each lot of antibody is quality control tested by immunofluorescent staining with flow cytometric analysis. Specificity testing of 1-3 target cell types with either single- or multi-color analysis (including positive and negative cell types). Once specificity is confirmed, each new lot must perform with similar intensity to the in-date reference lot. Brightness (MFI) is evaluated from both positive and negative populations. Each lot product is validated by QC testing with a series of titration dilutions.

Staining antibodies from BD Biosciences:
-PE Rat Anti-Mouse CD274: the MIH5 antibody reacts with CD274, also known as B7-H1 or PDL1. QC tested for mouse reactivity. Routinely Tested for Flow cytometry.
-BV650 Rat Anti-Mouse CD8a: the 53-6.7 monoclonal antibody specifically binds to the 38 kDa α and 34 kDa α' chains of the CD8 differentiation antigen (Ly-2 or Lyt-2) of all mouse strains tested. Routinely Tested for Flow cytometry.
-BV711 Rat Anti-Mouse CD4: tested in Development for mouse reactivity. The RM4-4 monoclonal antibody specifically binds to CD4 (L3T4) differentiation antigen expressed on most thymocytes, a subpopulation of mature T lymphocytes (ie, MHC class II-restricted T cells, including most T helper cells), and a subset of NK-T cells of all mouse strains tested. Qualified for Flow cytometry.

Primary antibodies from Cell Signaling Technologies:
CST™ antibodies are produced in-house and validated extensively according to a rigorous protocol.
Validation Steps Include:
-Examination of several cell lines and/or tissues of known expression levels allows accurate determination of species cross-reactivity and verifies specificity.
-Treatment of cell lines with growth factors, chemical activators or inhibitors, which induce or inhibit target expression, verifies specificity. Phosphatase treatment confirms phospho-specificity.
-The use of siRNA transfection or knockout cell lines verifies target specificity.
-Side-by-side comparison of lots to ensures lot-to-lot consistency.
-Optimal dilutions and buffers are predetermined, positive and negative cell extracts are specified, and detailed protocols are already optimized, saving valuable time and reagents.

Unconjugated human PD-L1 antibody (eBioscience): the MIH1 monoclonal antibody reacts with human B7-H1, also known as PD-L1. The MIH1 antibody has been tested by flow cytometric analysis of normal human peripheral blood cells.

Eukaryotic cell lines

Policy information about cell lines

Cell line source(s)

A549 (CCL-185), H460 (HTB-177), H1437 (CRL-5872), and H1944 (CRL-5907) NSCLC cells were purchased from ATCC and maintained in RPMI media with 10% fetal bovine serum (FBS). Mouse cancer cell lines KLN205 (CRL-1453), B16F10 (CRL-6475), and 4T1 (CRL-2539) were purchased from ATCC and maintained in EMEM + 10% FBS, DMEM + 10% FBS, and RPMI + 10% FBS, respectively. Lewis Lung Carcinoma (LLC) metastatic variant, LLC-JSP cells were gift from Dr. Don Gibbons lab (MD Anderson Cancer Center), and were cultured in RPMI + 10% FBS. BMDCs were harvested from naïve mice and cultured following published protocols.

Authentication

ATCC cell lines authentication:
-STR analysis (DNA profiling) for intraspecies identification and authentication of human cell lines
-Cytochrome C Oxidase subunit 1 (COI) for interspecies identification

Gifted cell line (LLC-JSP) was not authenticated.

Mycoplasma contamination

ATCC cell lines tested for mycoplasma contamination:
-Hoechst DNA stain (indirect)
-Agar culture (direct)

LLC-JSP cells were not tested for mycoplasma contamination.

Commonly misidentified lines
(See ICLAC register)

No commonly misidentified cell lines were used in this study.

Animals and other organisms

Policy information about studies involving animals; ARRIVE guidelines recommended for reporting animal research

Laboratory animals	6-week old NCI C57BL/6NCr (catalog #556) and DBA/2 (catalog #026) female mice were from the Charles River NCI Colony. Mice were housed in the Division of Comparative Medicine facilities at OHSU under 12:12 hr light:dark cycles, 20-25°C, and 30-70% humidity.
Wild animals	The study did not involve wild animals.
Field-collected samples	The study did not involve samples collected from the field.
Ethics oversight	All studies were reviewed and approved by Institutional Animal Care and Use Committee (IACUC) at Oregon Health and Science University (OHSU)

Note that full information on the approval of the study protocol must also be provided in the manuscript.

Flow Cytometry

Plots

Confirm that:

- [x] The axis labels state the marker and fluorochrome used (e.g. CD4-FITC).
- [x] The axis scales are clearly visible. Include numbers along axes only for bottom left plot of group (a 'group' is an analysis of identical markers).
- [x] All plots are contour plots with outliers or pseudocolor plots.
- [x] A numerical value for number of cells or percentage (with statistics) is provided.

Methodology

Sample preparation	For in vitro studies, cells were plated in 6-well plates and treated with indicated treatments. 3 days post treatments, cells were collected and washed in FACS buffer prior to staining. Primary (unconjugated or fluorophore-conjugated) and secondary antibodies were stained for 30 mins and 1 hour, respectively, under rocking on ice. After staining, cells were washed in FACS buffer before analysis with Guava easyCyte (Millipore Sigma) flow cytometer (10,000 events per sample). For immune profiling, tumors and tumor-draining lymph nodes were harvested and cut into small sections for digestion. Tissues were digested in digestion media (1 mg mL–1 Collagenase D and 0.1 mg mL–1 DNase I in HBSS) at 37 °C for 30 min and mechanically dissociated by passing through 70 µm pore nylon cell strainers. Red blood cells in the sample were lysed by incubating in RBC lysis buffer (Alfa Aesar) at room temperature for 5 min. Cells were washed twice with PBS and stained with Live/Dead Fixable Aqua Stain (Thermo Fisher Scientific) for 15 min. Cells were washed twice with FACS buffer (1% BSA in PBS), incubated with FcR blocking solution for 5 min, and then stained for a select panel of surface-staining antibodies for 15 min at room temperature. Intracellular staining (for FoxP3 and Ki67) was performed with BD Cytofix/Cytoperm (BD Biosciences), following the manufacturer's protocol after cell surface staining. Samples were washed twice with FACS buffer and resuspended in FACS buffer for analysis. All data were acquired with a BD LSRFortessa flow cytometer (OHSU's Flow Cytometry Core), and analyzed using FlowJo Software (TreeStar Inc.). Only live cells (determined by live–dead stain occurring before fixing/permeabilization) were analyzed.
Instrument	Guava EasyCyte; BD LSRFortessa flow cytometer
Software	All flow cytometry data was analyzed using FlowJo software.
Cell population abundance	For in vitro studies, at least 10,000 cells per sample were collected. For in vivo studies, at least 100,000 cells per sample were collected.
Gating strategy	Single viable cells identified in FSC/SSC gating to remove cell debris and live-dead staining. The population of immune cells gated in CD45-APC/Cy7. The population of CD8+ T cells (BV650) was gated as CD45+CD3+CD8+. The population of CD4+ T cells (BV711) was gated as CD45+CD3+CD4+. The population of proliferative effector T cells gated as Ki67+CD44+CD45+CD3+CD8+. Regulatory T cells (Tregs) gated as CD45+CD3+CD4+FoxP3+. PD-L1 expression presented as median fluorescent intensity (MFI) in gated CD45+ and CD45- cells.

- [x] Tick this box to confirm that a figure exemplifying the gating strategy is provided in the Supplementary Information.
